# Specificity of expression of *TaCKX* family genes in developing plants of wheat and their co-operation within and among organs

**DOI:** 10.1371/journal.pone.0214239

**Published:** 2019-04-10

**Authors:** Hanna Ogonowska, Karolina Barchacka, Sebastian Gasparis, Bartosz Jablonski, Waclaw Orczyk, Marta Dmochowska-Boguta, Anna Nadolska-Orczyk

**Affiliations:** 1 Department of Functional Genomics, Plant Breeding and Acclimatization Institute–National Research Institute, Radzikow, Blonie, Poland; 2 Department of Genetic Engineering, Plant Breeding and Acclimatization Institute–National Research Institute, Radzikow, Blonie, Poland; ICAR-Indian Institute of Agricultural Biotechnology, INDIA

## Abstract

Multigene families of *CKX* genes encode cytokinin oxidase/dehydrogenase proteins (CKX), which regulate cytokinin content in organs of developing plants. It has already been documented that some of them play important roles in plant productivity. The presented research is the first step of comprehensive characterization of the bread wheat *TaCKX* gene family with the goal to select genes determining yield-related traits. The specificity of expression patterns of fifteen formerly annotated members of the *TaCKX* family was tested in different organs during wheat development. Based on this, the genes were assigned to four groups: *TaCKX10*, *TaCKX5* and *TaCKX4*, highly specific to leaves; *TaCKX3*, *TaCKX6* and *TaCKX11*, expressed in various levels through all the organs tested; *TaCKX1*, *TaCKX2*.*3*, *TaCKX2*.*2*, *TaCKX2*.*1*, *TaCKX2*.*4* and *TaCKX2*.*5* specific to developing spikes and inflorescences; *TaCKX9*, *TaCKX8* and *TaCKX7*, highly specific to roots. Amplification products of tested genes were mapped to the chromosomes of the A, B or D genome using *T*. *aestivum* Ensembl Plants. Based on analysis of *TaCKX* transcripts as well as encoded amino acids in *T*. *aestivum* and *Hordeum vulgare* the number of *CKX* genes in wheat was limited to 11 and new numbering of selected *TaCKX* genes was proposed. Moreover, we found that there were developmental differences in expression of *TaCKX* in the first and the second spike and expression of some of the genes was daily time dependent. A very high and significant correlation was found between expression levels of *TaCKX7* and *TaCKX9*, genes specific to seedling roots, *TaCKX1*, *TaCKX2*.*1* and *TaCKX2*.*2*, specific to developing spikes, and the group of *TaCKX3*, *4*, *5*, *6*, *10* and *11*, highly expressed in leaves and other organs. The genes also co-operated among organs and were included in two groups representing younger or maturating stages of developing plants. The first group was represented by seedling roots, leaves from 4-week old plants, inflorescences and 0 DAP spikes; the second by developing spikes, 0 DAP, 7 DAP and 14 DAP. The key genes which might determine yield-related traits are indicated and their possible roles in breeding strategies are discussed.

## Introduction

Bread/common wheat is a very important crop in global agriculture because its grains are a worldwide staple food. Its productivity is high, but rising consumption and changing climate indicate the need of further improvement in yield potential [1]. The genome of this allohexaploid species is complex (2n = 6x = 42), composed of three homologous, diploid genomes (AABBDD). It is very large (1.7 x 10^10^ bp/1C), which makes genetic and molecular research as well as breeding very challenging. Recent development of advanced biotechnology tools indicated the role of specific key genes in wheat and barley for plant productivity (reviewed in Nadolska-Orczyk et al. [2]). Among those listed are *CKX* genes. *CKX* genes form multigene families in different plant species. In allohexaploid bread wheat (*Triticum aestivum* L.) 11 *TaCKX* genes have been identified so far and numbered from *TaCKX1* to *TaCKX11* (NCBI databases) and five *TaCKX2* (*TaCKX2*.*1* to *TaCKX2*.*5*), which have undergone a Triticeae-specific gene-duplication event [3]. *TaCKX1* and *TaCKX5* have been cloned by Feng et al. [4] and by Lei et al. [5]. Both *TaCKX2*.*1* (FJ648070) and *TaCKX2*.*2* (GUO84177) were isolated and characterized by Zhang et al. [6], and *TaCKX2*.3, *TaCKX2*.*4* and *TaCKX2*.*5* by Mameaux et al. [3]. Cloning and preliminary characterization of *TaCKX3* were done by Ma et al. [7]. The genes encode cytokinin oxidase/dehydrogenase proteins (CKX), which irreversibly degrade cytokinins [8–10], and thereby regulate cytokinin content in developing plants. Their expression is tissue-/organ- and developmentally-specific, suggesting genes’ specific functions [11, 12]. The detailed biological function of most of the *TaCKX* genes in wheat is not known.

Cytokinins (CKs) are known to be key regulators of seed yield in plants [13]. They act locally or alternatively they function as long distance signaling messengers [14] mediating and modulating sink strength [15–17]. The level of CKs in developing tissues/organs is regulated by balancing biosynthesis and degradation, and the CKX enzymes possibly play the principal role in this regulation [18–20]. The important role of selected *CKX* genes in cereal productivity has already been documented. In rice, loss-of-function mutation of *OsCKX2* caused an elevated cytokinin level, leading to an increase in reproductive organ number and seeds [21]. The same was also evidenced by RNAi silencing of the gene expression. In barley, decrease of expression of *HvCKX1* and *HvCKX9* by RNAi gene silencing in developing kernels and seedling roots led to an increase of seed and spike number, as well as plant productivity [12, 22, 23]. There are also two known examples of *TaCKX* variation in wheat leading to higher productivity. In the first one the haplotype variant of *TaCKX6-D1* was associated with a higher filling rate and grain size [24, 25]. In the second, the QTL found in recombinant inbred lines (RILs) containing a higher copy number of *TaCKX4* was associated with higher chlorophyll content and grain size [26].

According to Zalewski et al. [12], expression patterns of *HvCKX* genes indicated their role in growth and reproductive development of barley. Thinking about *CKX* regulation in wheat we started with the same hypothesis, which had been positively verified for barley. The goal of this research was to select those *TaCKX* genes which might regulate yield-related traits in wheat.

This first-step study on the role of *TaCKX* genes in plant development and productivity of wheat presents results on the specificity of the genes’ expression in various organs during wheat plant development and their co-operation. We also tested daily time dependency and spike number dependency of expression levels of the tested genes. In our forthcoming papers we will present phenotypic consequences of the observed *TaCKX* expression patterns for yield-related traits in breeding materials as well as the detailed roles of *TaCKX1* and *TaCKX2*.*1/TaCKX2*.*2* based on RNAi silenced wheat lines.

## Materials and methods

### Plant material

The experimental tissue samples were collected from three cultivars of common wheat (*Triticum aestivum* L.): Kontesa, Ostka and Trappe. Ten seeds from each cultivar were germinated into Petri dishes for one day at 4°C and then five days at room temperature in the dark. Six out of ten seedlings from each Petri dish were replanted into pots with soil. The plants were grown in a growth chamber under controlled environmental conditions with 20°C/18°C day/night temperatures and a 16 h light/8 h dark photoperiod. The light intensity was 350 μmol· s^-1^·m^-2^. Plants were irrigated three times a week and fertilized once a week with Florovit according to the manufacturer’s instructions.

The following tissue samples in three biological replicates from each cultivar were collected: 5-day-old seedlings roots, well-developed leaves from 4-week-old plants (the longest leaf); 5–6 cm long inflorescences and spikes: 0 days after pollination (0 DAP), first spike 7 DAP (I), second spike 7 DAP (II) and 14 DAP. All these samples were collected at 9:00 am. Additionally, the first 7 DAP spikes were collected at three time points: 9 am, 12 pm and 3 pm. The collected material was frozen in liquid nitrogen and kept at -80°C until use.

### RNA extraction and cDNA synthesis

Total RNA from all collected tissues was extracted using TRI Reagent (Sigma-Aldrich) according to the manufacturer’s protocol. The purity and concentration of the isolated RNA were determined using a NanoDrop spectrophotometer (NanoDrop ND-1000) and the integrity was checked by electrophoresis on 1.5% (w/v) agarose gels. To remove the residual DNA the RNA samples were treated with DNase I, RNase-free (Thermo Fisher Scientific). Each time 1 μg of good quality RNA was used for cDNA synthesis using the RevertAid First Strand cDNA Synthesis Kit (Thermo Fisher Scientific) following the manufacturer’s instructions. The obtained cDNA was diluted 20 times before use in RT-qPCR assays.

### Quantitative RT-qPCR

RT-qPCR assays were performed for 15 target genes. Primer sequences designed for each gene are shown in **S1 Table**. All real-time reactions were performed in a Rotor-Gene Q (Qiagen) thermal cycler using 1x HOT FIREPol EvaGreen qPCR Mix Plus (no ROX) (Solis BioDyne), 0.2 μM of each primer, and 4 μl of cDNA in a total volume of 10 μl. Each reaction was carried out in 3 biological and 3 technical replicates at the following temperature profile: 95°C– 15 min initial denaturation and polymerase activation (95°C– 25 s, 62°C– 25 s, 72°C– 25 s) x 45 cycles, 72°C– 5 min, with melting curve at 72–99°C 5 s per step. The expression of *TaCKX* genes was calculated according to the two standard curves method using ADP-ribosylation factor as a normalizer.

Relative expression was related to mean expression of *TaCKX3* measured in all organs and set as 1.00. Additionally, all data were counted in relation to the organ correction factor (OCR), which was the quotient of Ct number of the reference gene in the cDNA sample divided by the average Ct number for all samples.

Statistical analysis was performed using Statistica 13 (StatSoft) software. The Kruskal-Wallis test was used to verify the significance of the relative expression differences at the confidence level p<0.05. Correlations coefficients were determined using correlation matrices (Pearson test).

### Sequence data and phylogenetic analysis

Homologous sequences were retrieved from Ensembl Plants database [27]. The alignments of TaCKX and HvCKX amino acid sequences was conducted using the MAFFT version 7 program [28, 29]. The maximum likelihood phylogenetic tree was done on alignments of full-length protein sequences using MEGA X performed with the JTT model and 1000 bootstrap replicates [30].

## Results

### The family of *TaCKX* genes in wheat

According to the NCBI database and references there are 15 *TaCKX* genes; 11 of them are numbered from *TaCKX1* to *TaCKX11* (**Table 1**). In the case of *TaCKX2* five duplicates (*TaCKX2*.*1* to *TaCKX2*.*5*) are identified [3, 6]. Specific primers for expression analysis were designed for all of them, including *TaCKX2* duplicates. Based on *T*. *aestivum* IWGSC assembly (Ensembl Plants [27]), the three groups of transcripts: *TaCKX2*.*1*, *TaCKX2*.*2*, *TaCKX2*.*4* and *TaCKX6D1*; *TaCKX2*.*3*, *TaCKX2*.*5* and *TaCKX6a02* as well as *TaCKX7* and *TaCKX8* were located to the same homeoparalogues. Suggested names are: *TaCKX2*.*2* for the first group, *TaCKX2*.*1* for the second and *TaCKX7* for the third as indicated in the Table 1. The amplified regions of the genes were located to the following chromosomes: *TaCKX1* to chromosomes 3A, 3B, 3D; *TaCKX2*.*2*, *TaCKX2*.*1* and *TaCKX4* to chromosomes 3B, 3D; *TaCKX5* to chromosome 3B. The *TaCKX6* amplification product is located to chromosomes 1A, 1B, 1D and these locations are different from the *TaCKX6-D1b* and *a* alleles, which is 3D [24] and from the *TaCKX6a02* allele located to the 3DS [25]. There is no pairwise homology among them. Investigated by us *TaCKX7* is located to chromosomes 6B and 6D, *TaCKX9* to chromosomes 7A, 7B and 7D, and *TaCKX11* to chromosome 2A. According to NCBI *T*. *aestivum* genome assembly the primers for *TaCKX3* gene amplification hybridized with chromosome 7B and for *TaCKX10* with 1A, 1B and 1C (not showed).

**Table 1:** Comparison of analysed wheat *TaCKX* gene family members published in NCBI with ensemble plants (IWGSC assembly) databases. Primers for amplification were designed based on accession numbers of the genes in NCBI (in bold).

Gene (suggested annotation in bold)	Former annotation	Accesion numer NCBI or Ensembl Plants	Chrom. location	No. of exons/ coding exons	Protein (aa)	Amplicon length (bp)	Refer. (query cover/ ident. %)
*TaCKX1*		**JN128583**	-	-	501	188	[31]
		TraesCS3A02G109500	3A	3	524	188	(100/98)
		TraesCS3B02G128700	3B	3	524	188	(100/98)
		TraesCS3D02G111300	3D	3	524	188	(100/98)
***TaCKX2*.*1*^1^**	*TaCKX2*.*3*^1^	**JF293079**	-	-	553	144	[3]
		TraesCS3A02G311000	3A	3	567	na	(87/91)
		TraesCS3B02G161100	3B	3	578	144	(94/95)
		TraesCS3D02G143600	3D	3	551	144	(97/100)
	*TaCKX2*.*5*^1^	**JN381556**	-	3	545	147	[3]
		TraesCS3A02G311000	3A	3	567	na	(41/91)
		TraesCS3B02G161100	3B	3	578	na	(41/100)
		TraesCS3D02G143600	3D	3	551	na	(41/97)
	*TaCKX6a02*^1^	NI	3D	-	-	na	[25]
		TraesCS3D02G143600	3D	3	551	na	(100/99)
***TaCKX2*.*2*^2^**	*TaCKX2*.*1*^2^	**FJ648070**	3D*	-	547	205	[6]
		TraesCS3A02G311100	3A	3	552	na	(91/92)
		TraesCS3B02G161000	3B	3	547	205	(99/99)
		TraesCS3D02G143300	3D	3	547	221	(96/95)
		TraesCS3D02G143500	3D	3	547	224	(100/94)
	*TaCKX2*.*2*^2^	**GU084177**	3D*	-	547	175	[6]
		TraesCS3A02G311100	3A	3	552	na	(96/92)
		TraesCS3B02G161000	3B	3	547	175	(97/95)
		TraesCS3D02G143300	3D	3	547	175	(100/99)
		TraesCS3D02G143500	3D	3	547	175	(97/95)
	*TaCKX2*.*4*^2^	**JN381555**	-	3	552	220	[3]
		TraesCS3A02G311100	3A	3	552	na	(53/100)
		TraesCS3B02G161000	3B	3	547	na	(53/94)
		TraesCS3D02G143300	3D	3	547	na	(53/94)
		TraesCS3D02G143500	3D	3	547	na	(53/94)
	*TaCKX6D1*^2^	JQ797673	3D*	3	545	155	[24]
		TraesCS3A02G311100	3A	3	552	na	(44/94)
		TraesCS3B02G161000	3B	3	547	na	(44/97)
		TraesCS3D02G143300	3D	3	547	na	(44/99)
		TraesCS3D02G143500	3D	3	547	na	(44/99)
*TaCKX3*		**JN128585**	-	-	-	150	[31]
		TraesCS7A02G536900	7A	3	516	na	(76/98)
		TraesCS7B02G455000	7B	4	516	na	(98/99)
*TaCKX4*		**JN128586**	-	-	-	112	[31]
		TraesCS3B02G525300	3B	5	525	111	(99/99)
		TraesCS3D02G475800	3D	5	525	112	(100/94)
*TaCKX5*		**NI**	3B	-	-	150	[5]
		TraesCS3A02G321100	3A	5	530	144	(95/95)
		TraesCS3B02G344600	3B	5	531	150	(98/100)
		TraesCS3D02G310200	3D	5	531	na	(90/96)
*TaCKX6*		**JN128587**	-	-	-	182	[31]
		TraesCS1A02G159600	1A	5	522	182	(99/97)
		TraesCS1B02G176000	1B	6/5	522	182	(94/98)
		TraesCS1D02G157000	1D	5	522	182	(100/99)
***TaCKX7*^3^**	*TaCKX7*^3^	**JN128588**	-	1	534	144	[31]
		TraesCS6A02G185800	6A	1	533	na	(100/96)
		TraesCS6B02G214700	6B	1	533	na	(100/96)
		TraesCS6D02G172900	6D	2	467	na	(87/97)
	*TaCKX8*^3^	**JN128589**	-	1	534	198	[31]
		TraesCS6A02G185800	6A	1	533	na	(100/96)
		TraesCS6B02G214700	6B	1	533	198	(100/99)
		TraesCS6D02G172900	6D	2	467	198	(87/96)
*TaCKX9*		**JN128590**	-	1	262	278	[31]
		TraesCS7A02G363400	7A	2	551	278	(100/95)
		TraesCS7B02G264400	7B	2	540	278	(100/99)
		TraesCS7D02G359700	7D	2	532	278	(98/96)
*TaCKX10*		**JN128591**	-	-	-	167	[31]
		TraesCS1A02G234800	1A	6/5	521	na	(96/99)
		TraesCS1B02G248700	1B	5	521	na	(96/99)
		TraesCS1D02G237200	1D	5	521	na	(96/99)
*TaCKX11*		**JN128592**	-	-	245	184	[31]
		TraesCS2A02G378300	2A	5	528	184	(99/97)

*—primary mapped

^1,2,3^ –located in the same gene respectively; NI—no identified;—no information; na–not amplified

The distance tree of pairwise comparison (**Figs A-N in S1 File**) revealed that homologues of *TaCKX1* were located on chromosomes 3A, 3B, 3D (*T*. *aestivum* genome assembly) with identity of 98% of whole sequences (100% cover). The gene is highly homologous to *Aegilops tauschii cytokinin dehydrogenase 1-like* (99% cover/98% identity), *Secale cereale ScCKX1* (45% cover/97% identity), *Hordeum vulgare CKX1* (100% cover/94% identity) and *Zea mays CKX1* (57% cover/84% identity). The *TaCKX2*.*2* isolated by Zhang et al. [6] is proved to be located on 3D and their homologue on 3B has 95% identity and on 3A 92% identity. The gene is highly homologous to *A*. *tauschii cytokinin dehydrogenase 2-like* (95% cover/96% identity), *H*. *vulgare CKX2*.*2* (93% cover/92% identity), *Z*. *mays CKX5* (83% cover/77% identity), and *Oryza sativa* (85% cover/79% identity). Moreover, *TaCKX2*.*2* is 91% identical (under 38% cover) to *S*. *cereale ScCKX2*.*2*. The third *TaCKX2*.*1*, for which primers were designed based on JF293079 [3] has highest cover/identity with 3D (97/100%) and lower with 3B (94/95%) and 3A (87/91%) *T*. *aestivum* Esembl Plants and is highly homologous to *A*. *tauschii cytokinin dehydrogenase 2-like* (96% cover/99% identity).

The primers for *TaCKX3* were designed based on the JN128585 sequence isolated by Song et al. [31] hybridized with NCBI LS992099 from *T*. *aestivum* NCBI genome assembly (not showed) and showed 99% identity with *TaCKX3* (GQ925404) isolated by Ma et al. [7]. The whole sequence located on chromosome 7B has 99% identity to the sequence from *T*. *aestivum* IWGSC assembly chromosome 7B and 98% identity with 76% cover with 7A. The closest orthologues among other cereal species are: *A*. *tauschii cytokinin dehydrogenase 11* (96% cover/95% identity), *S*. *cereale ScCKX11* (72% cover/95% identity), *H*. *vulgare* (64% cover/93% identity), *Z*. *mays CKX10* (74% cover/86% identity), and *O*. *sativa* (75% cover/87% identity).

The primers for *TaCKX4*, isolated by Song et al. [31] and deposited in NCBI as an unverified accession, hybridized with the closest homologue found on chromosome 3B *T*. *aestivum* IWGSC assembly (99% cover/99% identity) and 3D (100% cover/94% identity). Close orthologues of the gene were *A*. *tauschii CKX4-like* showing 94% identity under 97% cover, *H*. *vulgare CKX4* with 89% identity under 98% cover and *Lolium perenne* with 85% identity and under 82% cover.

Homologous copies of *TaCKX5* isolated by Lei et al. [5] have been localised on chromosomes 3A, 3B and 3D, however primers hybridized with 3B and the only amplification product was 150 bp long. Closest orthologues were *A*. *tauschii* with 96% identity and 90% cover, *S*. *cereale* with 96% identity and 73% cover, *O*. *sativa CKX5-like*, *Sorghum bicolor CKX5*, *Z*. *mays CKX4b*, *Panicum hallii CKX5* and *Setaria italica CKX5*, all with identity 77–78% and 78–80% cover.

*TaCKX6* analysed in this paper was isolated by Song et al. [31] and based on *T*. *aestivum* IWGSC assembly mapped to chromosomes 1A, 1B and 1D. In Table 1 two additional, described in the literature, *TaCKX6* genes were included: *TaCKX6-D1b* [24] and *TaCKX6a02* [25]. However transcript of the first one was located to the *TaCKX2*.*2* and the second to the *TaCKX2*.*1* of chromosome 3D. The closest homologue of *TaCKX6* was *T*. *aestivum CKX8* sharing 98% identity under 89% cover of the sequences. Very close orthologues were: *H*. *vulgare CKX3* with 94% identity, *L*. *perenne CKX6* with 96% identity and *Z*. *mays CKX6* sharing 81% identity under 83% cover. The closest homologues of *TaCKX-D1b* and *a* alleles was *TaCKX2*.*2* sharing 98% identity under 43% cover. Their close orthologues were *A*. *tauschii CKX2-like* with 99% identity and 43% cover and *H*. *vulgare CKX2*.*2* with 88% identity under 69% cover. The closest homologue of *TaCKX6a02* was *T*. *aestivum CKX2*.*1* with 99% identity under 100% cover.

The sequences of *TaCKX7* and *TaCKX8* isolated by Song et al. [31] were located to the same *TaCKX7* gene and their primers hybridized with 6B and 6D, and shared 100% cover with chromosomes 6A and 6B, however with lower, 96% identity. Very close orthologues were: *A*. *tauschii CKX6-like* with 96% similarity under 100% cover, *H*. *vulgare CKX7* with 99% cover and 90% identity, and *Z*. *mays CKX7*, *S*. *bicolor CKX7*, *S*. *italica CKX7*, *P*. *halli CKX7*, all with 77–78% identity under 93–95% cover.

Other *TaCKX* genes isolated by Song et al. [31] and analysed in this paper are *TaCKX9*, *TaCKX10* and *TaCKX11*. Based on *T*. *aestivum* IWGSC assembly primers for the first one hybridized to chromosomes 7A, 7B and 7D. The second one was *in silico* amplified on chromosomes 1A, 1B and 1D, according to NCBI *T*. *aestivum* genome assemble (not showed) but not to IWGSC assembly. Close orthologues to *TaCKX9* were: *H*. *vulgare CKX10* showing 95% identity under 71% cover, *A*. *tauschii cytokinin dehydrogenase 10-like* (99% cover/97% identity), and *Z*. *mays CKX10*, *S*. *italic CKX10* and *P*. *halli CKX10* with 83–84% identity and under 95–96% cover. The *TaCKX10* sequence had 100% cover and 99% identity to chromosomes 1A, 1B and 1D according to NCBI *T*. *aestivum* genome assemble but 99% cover and 96% identity according to IWGSC assembly. Close homologues to *TaCKX10* were: *A*. *tauschii cytokinin dehydrogenase 9* (96% cover/99% identity), *H*. *vulgare CKX3* (96% cover/99% identity), *H*. *vulgare CKX2* (98% cover/96% identity), which was further annotated as *HvCKX9* [3], *Brachypodium distachyon CKX9* (96% cover/90% identity), *O*. *brachyantha CKX9* (96% cover/85% identity), *O*. *sativa* chromosome 5 (97% cover/86% identity), *O*. *sativa CKX9* (96% cover/86% identity), *Sorghum bicolor CKX9* (95% cover/83% identity), *Setaria italica CKX9* (95% cover/84% identity).

The *TaCKX11* sequence had 99% cover and 97% identity with the corresponding region of chromosome 2A. Very close orthologues of the gene were: *A*. *tauschii CKX8-like* with 95% identity, under 99% cover and *H*. *vulgare CKX8* (81% cover/95% identity), *B*. *distachyon CKX8-like* (97% cover/86% identity), *O*. *sativa CKX8* (100% cover/83% identity), *P*. *hallii CKX8-like* (95% cover/82% identity), *S*. *italica CKX8* (97% cover/83% identity).

### Phylogenetic comparison of proteins encoded by *TaCKX* and *HvCKX*

The proteins encoded by *TaCKX2*.*3*, *TaCKX2*.*5* and *TaCKX6a02* grouped together in close association with the protein encoded by *HvCKX2*.*1* (**S2 Table and S1 Fig).** Hence the genes were assigned as *TaCKX2*.*1*. Another group composed of TaCKX2.1, TaCKX2.2, TaCKX2.4 and TaCKX6D1 was associated with HvCKX2.2. Based on this four wheat genes coding these proteins were assigned as *TaCKX2*.*2*. The proteins encoded by *TaCKX7* and *TaCKX8* grouped together in association with the protein encoded by *HvCKX7*. Hence the genes were assigned as *TaCKX7*.

### The *TaCKX* genes are more or less specifically expressed in different tissues during plant development

**Fig 1:**
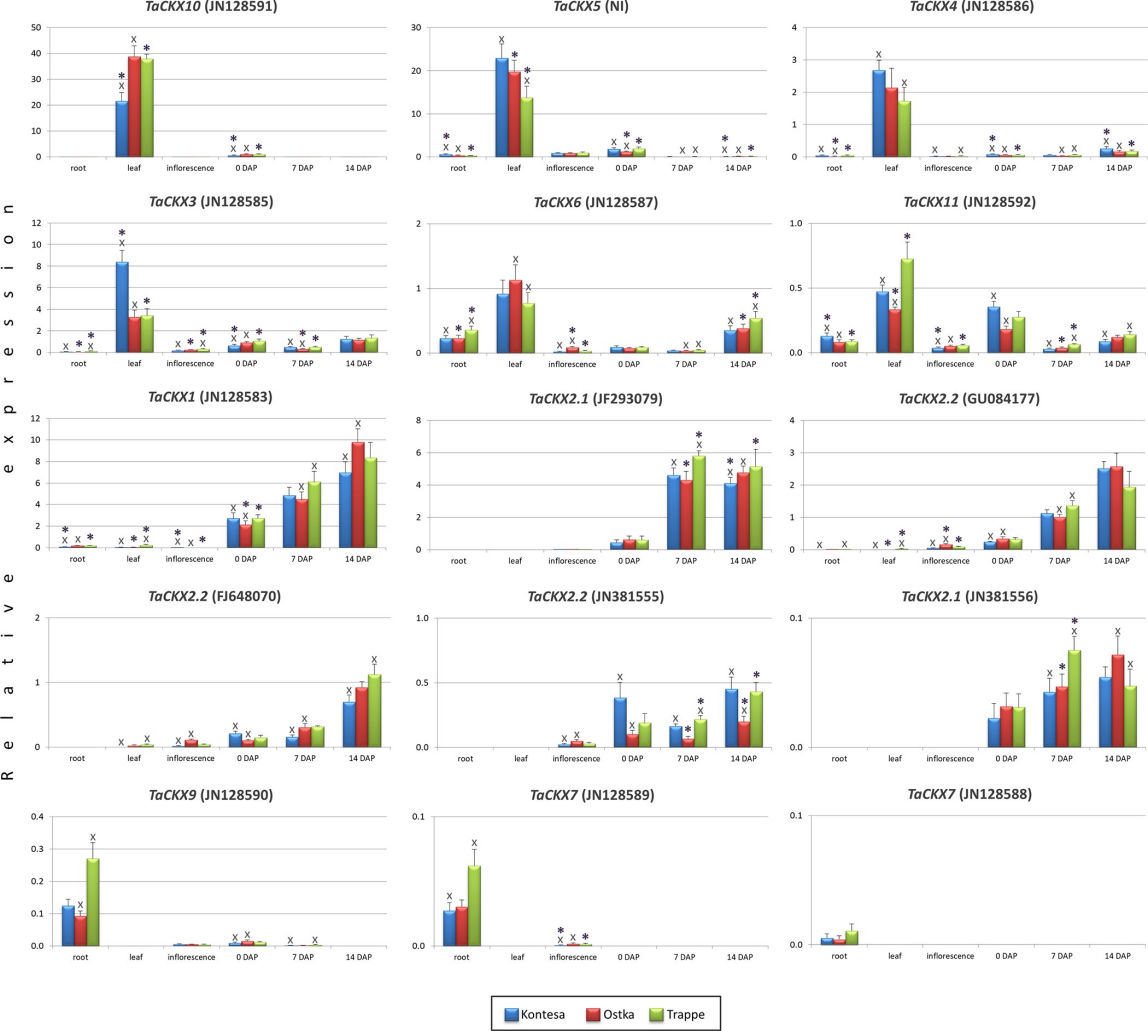
Specificity of *TaCKX* family gene expression in developing tissues/organs. The genes are annotated as suggested in Table 1 with the corresponding NCBI accession number in brackets. The relative expression of the genes was measured in: seedling root, well-developed leaf from 4-week-old plant, inflorescence, 0 DAP, 7 DAP and 14 DAP spikes in three cultivars, Kontesa, Ostka, Trappe. The level of expression was related to the mean expression of *TaCKX3* in all organs tested set as 1.00. The order of the genes is from the highest to the lowest expression, taking into consideration the specificity of expression. *–significant differences at P<0.05.

Specificity of *TaCKX* genes’ expression was tested in 5-day-old seedling roots, well-developed leaves from 4-week old plants, 5–6 cm long inflorescences, and 0 DAP, 7 DAP, 14 DAP spikes. The expression data are shown as relative values related to the mean expression of *TaCKX3* in all organs tested designated as 1.0. The most highly and specifically expressed gene was *TaCKX10* (**Fig 1**). The relative expression level of the gene ranging from 21.54 to 38.75 in well-developed leaves of 4-week-old plants depended on the cultivar tested. The differences in expression between cv. Kontesa and cvs. Ostka and Trappe were significant. The gene was also expressed in 0 DAP spikes with about a 40 times lower level compared to leaves (0.63–1.04).

The next gene in order of expression level was *TaCKX5*, which showed the highest expression in well-developed leaves (13.78–22.90) and then in 0 DAP spikes (1.19–1.90), inflorescence (0.88–0.96) and seedling roots (0.33–0.60) depending on the cultivar. There were significant differences in expression of the gene among cultivars in seedling root, leaf and 0 DAP spike.

*TaCKX1* was highly and specifically expressed in developing spikes with the highest expression level in 14 DAP spikes ranging from 6.96 in cv. Kontesa to 9.79 in cv. Ostka through 4.49 to 6.11 in 7 DAP spikes and from 2.11 in cv. Ostka to 2.73 in cvs. Kontesa and Trappe in 0 DAP spikes. Very low expression, below 0.2, was found in seedling roots and leaves. There were significant differences in expression of *TaCKX1* in developing spikes as well in seedling roots among the cultivars.

Interestingly, *TaCKX3* was expressed in all organs tested. The highest expression level of the gene was in leaves, ranging from 3.28 to 3.43 in cvs. Ostka and Trappe and to more than twice as high (8.39) in cv. Kontesa. Similarly, the relative expression level was above 1.0 in 14 DAP spikes and there were no significant differences among cultivars, and in 0 DAP spikes of cv. Trappe. There was a lower expression level in 7 DAP spikes (0.31–0.49), inflorescence (0.19–0.31) and the lowest in the seedling roots (0.05–0.12). Significant differences in expression levels among cvs. Kontesa, Ostka and Trappe were found in all organs tested excluding 14 DAP.

*TaCKX2*.*1* (JF293079) was highly and specifically expressed in developing spikes with the highest expression level in 7 DAP and 14 DAP spikes ranging from 4.11 in 14 DAP cv. Kontesa to 5.80 in 7 DAP cv. Trappe. There were significant differences in level of expression among the cultivars. Low expression (0.43–0.61) was found in 0 DAP spikes.

*TaCKX4*, *TaCKX2*.*2*(FJ648070), *TaCKX2*.*2*(GU084177), *TaCKX2*.*2*(JN381555), *TaCKX2*.*1*(JN381556), *TaCKX6*, *TaCKX11*, *TaCKX9*, *TaCKX7*(JN128589) and *TaCKX7*(JN128588) were low-expressed genes. The highest expression of the first one was in leaves (1.73–2.68). The gene was also expressed in other tested organs although at a very low level, from 0.01 in roots of cv. Ostka to 0.26 in 14 DAP spikes of cv. Kontesa. There were significant differences among the cultivars for almost all genes tested. *TaCKX2*.*2*(FJ648070), *TaCKX2*.*2*(GU084177), *TaCKX2*.*2*(JN381555) and *TaCKX2*.*1*(JN381556) were specifically expressed in generative organs: inflorescences and developing spikes, with much higher expression values in the second one. The relative expression of *TaCKX2*.*2*(FJ648070) in inflorescence ranged from 0.02 in cv. Kontesa to 0.11 in cv. Ostka, while for *TaCKX2*.*2*(GU084177) it was from 0.05 in cv. Kontesa to 0.16 in cv. Ostka. The expression patterns of *TaCKX2*.*2*(FJ648070) varied in 0 DAP spikes, showing the highest level in cv. Kontesa (0.21) and the lowest in cv. Ostka (0.10). Relative expression of the gene in 7 DAP spikes was about 3 times higher in cvs. Ostka and Trappe, but it was lower in cv. Kontesa. The highest expression of *TaCKX2*.*2*(FJ648070) observed in 14 DAP spikes ranged from 0.70 in cv. Kontesa to 1.12 in cv. Trappe. There were significant differences in the level of expression between cv. Kontesa and the two other cultivars.

The expression of *TaCKX2*.*2*(GU084177) in 0 DAP spikes was from 0.24 in cv. Kontesa up to 0.34 in cv. Ostka. In 7 DAP spikes the expression was about 4 times higher and in 14 DAP spikes about 8 times higher compared to 0 DAP spikes. There were significant differences in expression in all organs excluding 14 DAP among cultivars tested. The expression of *TaCKX2*.*2*(JN381555) and *TaCKX2*.*1*(JN381556) was very low, below 0.5 in developing spikes for the first one and below 0.1 for the second.

*TaCKX6* was expressed in all organs tested, showing a higher expression level in leaves (0.77–1.13) and about twice as low in 14 DAP spikes (0. 35–0.54). Interestingly, expression level of the gene was relatively high in the roots, ranging from 0.23 in cvs. Kontesa and Ostka to 0.36 in cv. Trappe. There were significant differences of the gene expression among cultivars in all organs tested excluding 0 DAP.

Similar to *TaCKX6*, *TaCKX11* was expressed in all organs tested, with expression levels ranging from 0.04 to 0.07 in inflorescences and 7 DAP spikes, about 0.1 in roots and 14 DAP spikes, 0.18 to 0.36 in 0 DAP spikes, up to 0.33–0.73 in leaves. There were significant differences of expression levels among cultivars in all organs.

*TaCKX9*, *TaCKX7*(JN128589) and *TaCKX7*(JN128588) were specifically expressed in roots showing low, from 0.09 in cv. Ostka to 0.27 in cv. Trappe and significant differences among cultivars for the first one; very low, from 0.03 to 0.06 for the second and minimal expression, below 0.012 for the third gene.

### There were differences between *TaCKX* expression levels in the first and the second spike

**Fig 2:**
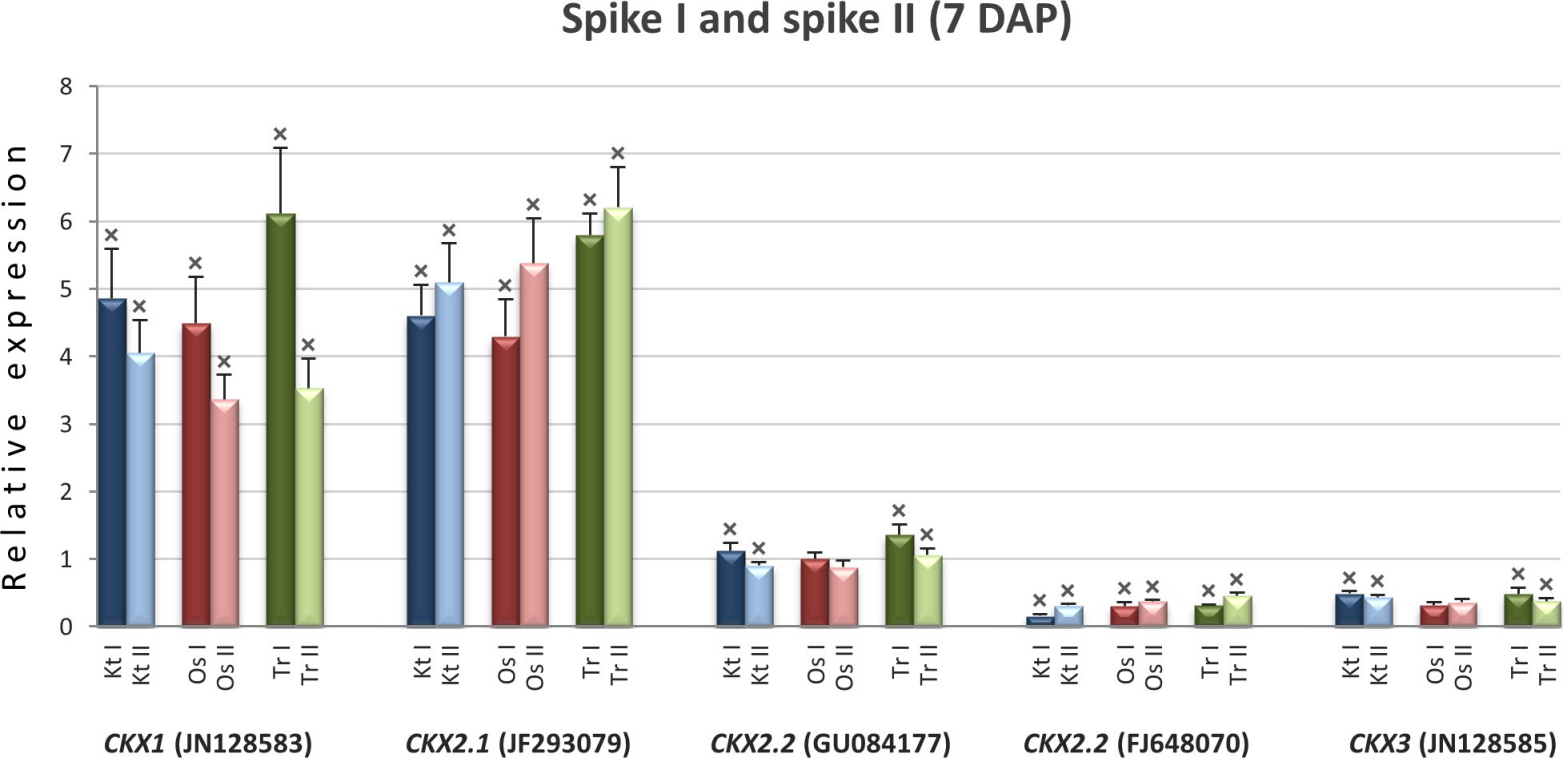
***TaCKX* gene expression in the first (I) and the second (II) developed 7 DAP spike in three cultivars, Kontesa, Ostka, Trappe.** The level of expression was related to the mean expression of *TaCKX3* set as 1.00. *–significant differences at P<0.05.

The differences in expression levels of *TaCKX* were measured in the first (I) and the second (II) 7 DAP spikes of plants from the three cultivars tested (**Fig 2**). There were significant differences in expression levels of I and II spikes in the case of *TaCKX1*, *TaCKX2*.*1*(JF293079) and *TaCKX2*.*2*(FJ648070) in the three cultivars tested and *TaCKX2*.*2*(GU084177) and *TaCKX3* in cvs. Kontesa and Trappe. Low expressing genes (*TaCKX4*, *TaCKX5*, *TaCKX6*, not showed) differ in expression in both spikes depending on the cultivar. In most cases expression levels of the genes in the I spikes were higher than in the II spikes. Opposite results were observed for *TaCKX2*.*1*(JF293079) and *TaCKX2*.*2*(FJ648070).

### *TaCKX* level of expression was daily time dependent

**Fig 3:**
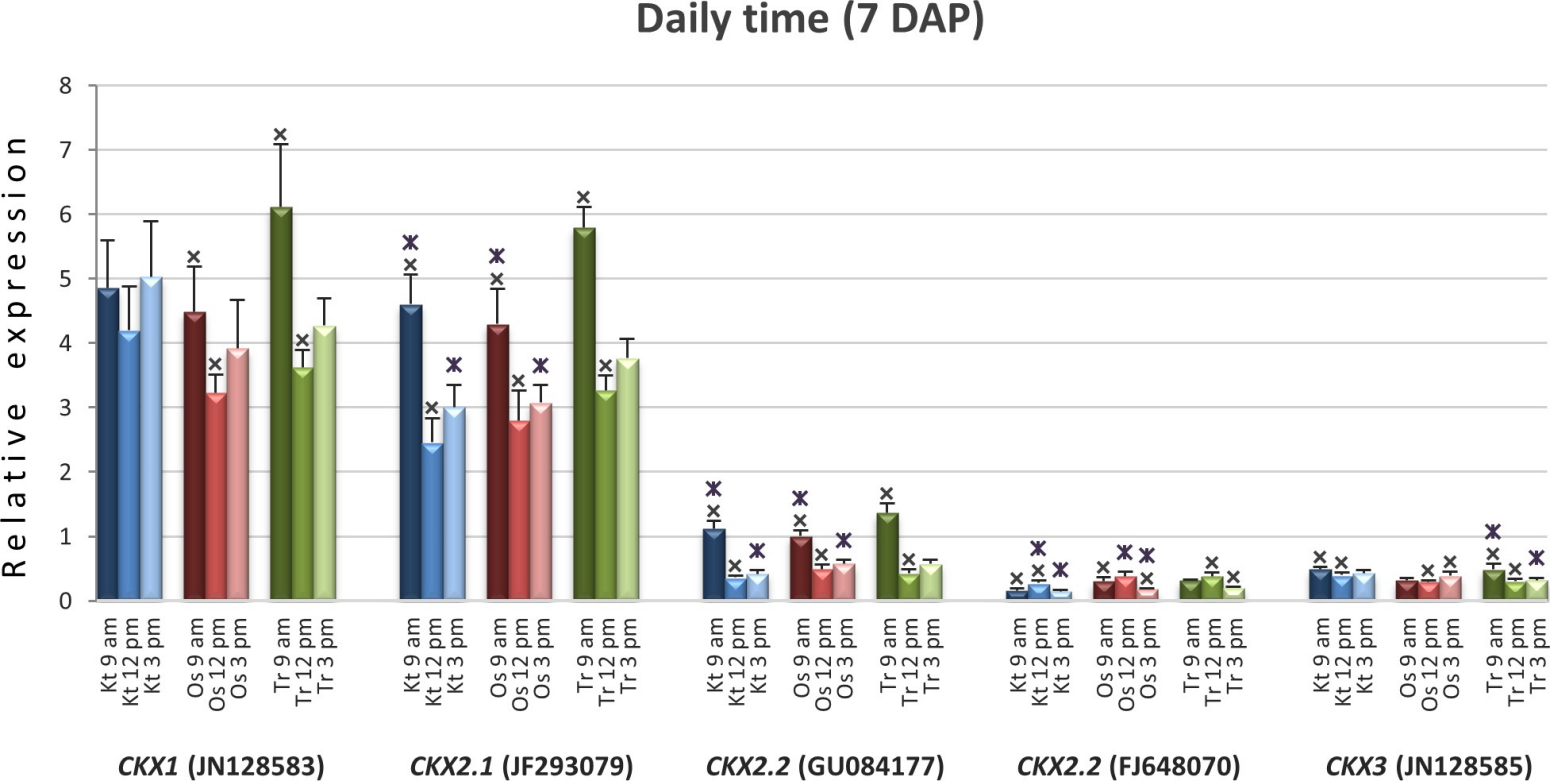
Daily time expression of *TaCKX* family genes in 7 DAP spikes for relatively high expressing genes. The first spikes were collected at 9:00 am, 12:00 pm and 3:00 pm from three cultivars, Kontesa, Ostka, Trappe. The level of expression was related to the mean expression of *TaCKX3* set as 1.00. *–significant differences at P<0.05.

Daily time dependence of expression level was measured in 7 DAP spikes I collected at 9:00 am, 12:00 pm and 3:00 pm in the three tested cultivars (**Fig 3**). There were significant differences among daily time expression for all tested *TaCKX* genes and in all three or two cultivars tested. No significant differences were observed for *TaCKX1* expression in cv. Kontesa and *TaCKX4* expression (not showed) in cv. Ostka.

### Co-operation of *TaCKX* genes among themselves and among organs

Correlation coefficients among *TaCKX* gene expression through investigated organs are presented in **Table 2**. The highest correlations were found between *TaCKX9*, *TaCKX7*(JN128589) and *TaCKX7*(JN128588) (0.99–1.0) and *TaCKX4* and *TaCKX5* (0.99). Very high coefficients were found in the group of *TaCKX3*, *TaCKX4*, *TaCKX5* and *TaCKX6* (0.79–0.93) and in the group of *TaCKX1*, *TaCKX2*.*2*(FJ648070) and *TaCKX2*.*2*(GU084177) (0.87–0.90) and between *TaCKX2*.*1*(FJ648070) and *TaCKX 2*.*1*(JN381556) (0.93). Slightly lower correlations from 0.55 to 0.83 were among *TaCKX1*and all clones of *TaCKX2*.*1* and *TaCKX2*.*2*. Similar level of correlation coefficients ranging from 0.71 to 0.91 were observed among *TaCKX11*, *TaCKX10* and *TaCKX3*, *4*, *5*, *6*. Moreover, *TaCKX6* correlated with *TaCKX3*, *TaCKX4*, *TaCKX5* at the level of 0.79, 0.89 and 0.87 respectively. There were also significant negative correlation coefficients between *TaCKX1* and *TaCKX5* at the level of -0.43 and *TaCKX1* and *TaCKX10* at -0.39. Besides, *TaCKX2*.*1*(FJ648070) and *TaCKX2*.*1*(JN381556) negatively correlated with *TaCKX5*, *10* and *11* at the level of -0.38 to -0.49.

**Table 2:** Correlations coefficients among *TaCKX* genes through investigated organs in three cultivars (N = 27). NCBI clones are in brackets.

	*CKX2*.*2* (FJ648070)	*CKX2*.*2* (GU084177)	*CKX2*.*1* (JF293079)	*CKX2*.*2* (JN381555)	*CKX2*.*1* (JN381556)	*CKX3*	*CKX4*	*CKX5*	*CKX6*	*CKX7* (JN128588)	*CKX7* (JN128589)	*CKX9*	*CKX10*	*CKX11*
***CKX1***	0.87**	0.90**	0.82**	0.70**	0.83**	no	-ns	-0.43*	no	-ns	-ns	-ns	-0.39*	-ns
***CKX2*.*2* (FJ648070)**		0.88**	0.70**	0.72**	0.71**	no	no	-ns	no	-ns	-ns	-ns	-ns	no
***CKX2*.*2* (GU084177)**			0.77**	0.71**	0.81**	no	no	-ns	no	-ns	-ns	-ns	-ns	-ns
***CKX2*.*1* (JF293079)**				0.55**	0.93**	-ns	-ns	-0.43*	-ns	-ns	-ns	-ns	-0.38*	-0.49*
***CKX2*.*2* (JN381555)**					0.64**	no	-ns	-ns	no	-ns	-ns	-ns	-ns	no
***CKX2*.*1* (JN381556)**						-ns	-ns	-0.43*	-ns	-ns	-0.39*	-ns	-0.40*	-0.39*
***CKX3***							0.93**	0.91**	0.79**	no	no	no	0.71**	0.72**
***CKX4***								0.99**	0.89**	no	no	no	0.91**	0.77**
***CKX5***									0.87**	no	no	no	0.91**	0.78**
***CKX6***										no	no	no	0.85**	0.71**
***CKX7* (JN128588)**											0.99**	1.00**	no	no
***CKX7* (JN128589)**												0.99**	no	no
***CKX9***													no	no
***CKX10***														0.81**

no—lack of correlation; ns—low and not significant

*- significant at p<0.05

**- significant at p<0.01

**Fig 4:**
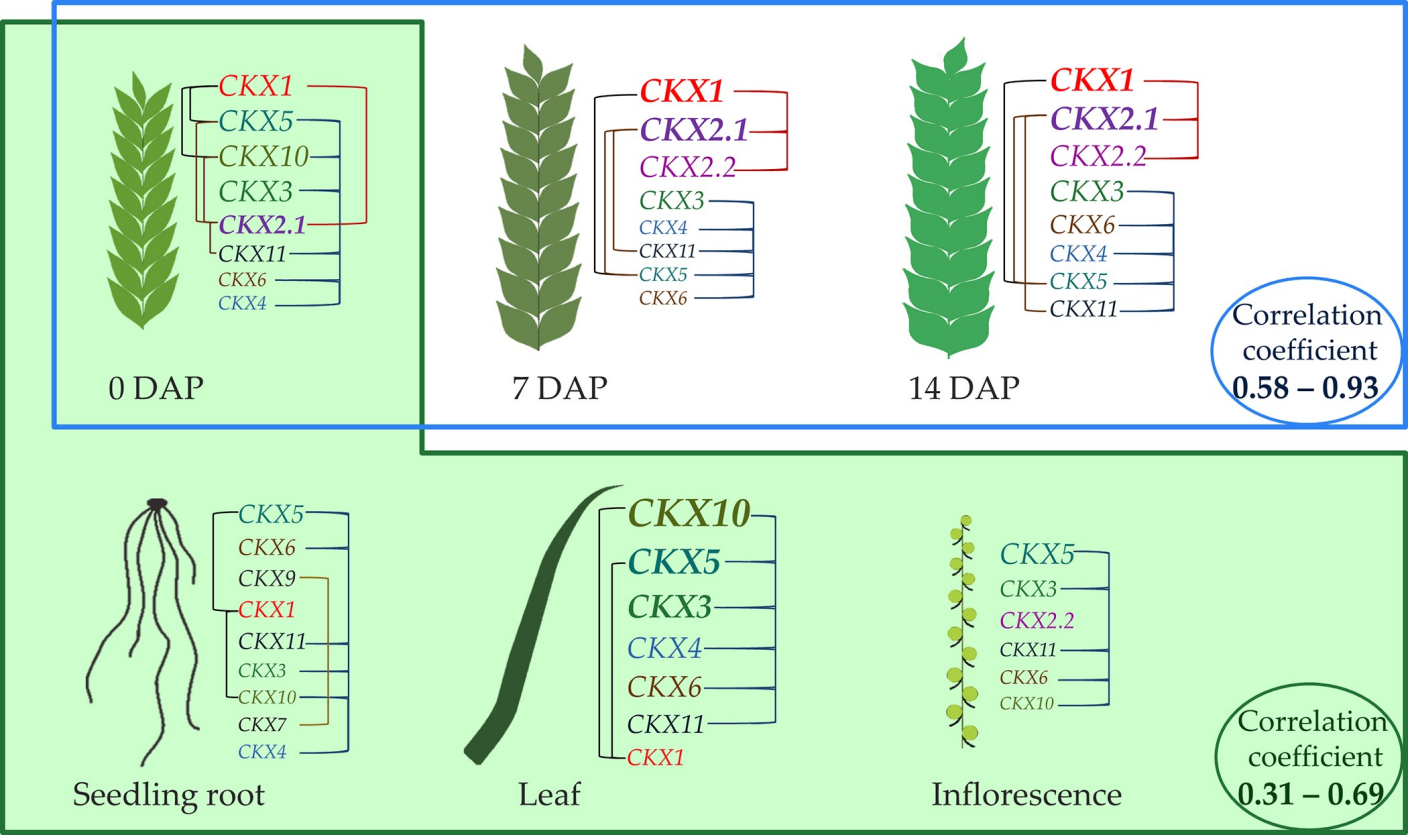
Graphic presentation of co-operation of *TaCKX* genes in the investigated organs in three cultivars tested. The genes with the highest expression in tested tissue/organ are at the top of the lists. Correlations of *TaCKX* gene expression within an organ are marked by lines and among organs by green and white boxes and blue line.

Correlation coefficients of *TaCKX* gene family expression among organs are presented in **Table 3** and a graphic presentation of co-operation of *TaCKX* genes in the investigated organs is shown in **Fig 4**. There were two groups of developing tissues/organs showing high correlation of *TaCKX* gene expression. The first one groups seedling roots, leaves from 4-week-old plants, inflorescences and 0 DAP spikes in which correlations of gene expression ranged from 0.31 to 0. 69 for the three cultivars tested. The second groups developing spikes, 0 DAP, 7 DAP and 14 DAP. In this group the lowest correlation coefficient for *TaCKX* gene expression was between 0 DAP and 7 DAP spikes (0.58), higher between 0 DAP and 14 DAP (0.66), and the highest between 7 DAP and 14 DAP (0.84).

**Table 3:** Correlations coefficients of *TaCKX* gene family among organs in three cultivars (N = 45).

	Leaf	Inflorescence	0 DAP	7 DAP	14 DAP
**Seedling root**	0.31*	0.69**	0.45**	no	no
**Leaf**		0.43**	0.35*	no	no
**Inflorescence**			0.44**	no	no
**0 DAP**				0.58**	0.66**
**7 DAP**					0.93**

no—lack of correlation

*- significant at p<0.05

**- significant at p<0.01

## Discussion

### New nomenclature of selected *TaCKX*

The nucleotide sequences of NCBI accessions: FJ648070, GU084177, JN381555 and JQ797673 previously annotated as *TaCKX2*.*1*, *TaCKX2*.*2*, *TaCKX2*.*4* and *TaCKX6D1* respectively, were located to the same homeoparalogues in wheat A, B and D subgenomes: TraesCS3A02G311100, TraesCS3B02G161000 TraesCS3D02G143300 and TraesCS3D02G143500. Proteins encoded by the genes were phylogenetically closely associated with the protein encoded by *HvCKX2*.*2* therefore our suggested name for these homeoparalogues is *TaCKX2*.*2*. Similarly, accessions JF293079 and JN381556 previously annotated as *TaCKX2*.*3* and *TaCKX2*.*5* were located, along with reported by [25] *TaCKX6a02*, to the same homeoparalogues TraesCS3A02G311000, TraesCS3B02G161100 and TraesCS3D02G143600. Proteins encoded by the genes were phylogenetically closely associated with the *HvCKX2*.*1* encoded protein. Hence, the wheat homeoparalogues were assigned as *TaCKX2*.*1*. The accessions JN128588 and JN128589, previously assigned as *TaCKX7* and *TaCKX8*, were highly similar to homeoparalogues TraesCS6A02G185800, TraesCS6B02G214700 and TraesCS6D02G172900. The genes-encoded proteins were phylogenetically close to HvCKX7, therefore our suggested name for the gene is *TaCKX7*. The similarities of proteins encoded by barley and wheat *CKX* genes as well as discussed below expression patterns and expression correlations of wheat *CKX* genes supported suggested by us numbering of *CKX* genes in wheat. The number of 11 *CKX* genes in wheat and barley are coherent for the both species.

### Specificity of expression of *TaCKX* family genes and phylogenic analysis

Considering the specificity of expression, the *TaCKX* genes can be assigned to four groups: *TaCKX10*, *TaCKX5* and *TaCKX4* were highly specific to leaves, *TaCKX1*, *TaCKX2*.*1* and *TaCKX2*.*2* to developing spikes, *TaCKX9* and *TaCKX7* to roots, and *TaCKX3*, *TaCKX6*, *TaCKX11* are more or less expressed through the all organs tested.

The first two genes from the group specific to leaves, *TaCKX10* and *TaCKX5* have the highest expression level among tested organs of developing wheat plants. Based on *T*. *aestivum* genome assembly data the amplification product of the *TaCKX10* gene isolated by Song et al. [31] was proved to be located to chromosomes 1A, 1B, 1D and was one of few *TaCKX*, which was most likely expressed from three homologous genomes. Function of the gene is not characterized. A very close homolog of *TaCKX10* found in databases is *H*. *vulgare CKX9*, formerly annotated as *HvCKX2*, and this gene has been previously characterized [12, 23, 32]. In the last report anti-HvCKX9 antibodies predominantly detected proteins in the leaf vasculature; however, according to Zalewski et al. [12] expression of *HvCKX9* was highest in 14 DAP kernels, 30-fold higher than in other tissues of two barley cultivars. Overexpression of the gene under a constitutive promoter caused very slow growth and plants died without flowering [32]. In contrast, the phenotypic result of *HvCKX9* silencing by stable, *Agrobacterium*-mediated RNAi was a higher number of seeds and higher grain yield [23]. Very close homology of *TaCKX10* with *HvCKX9* (96% identity under 98% cover) and the proteins encoded by these genes might suggest similar functions; however, expression patterns of these orthologues, although measured in the same organs during wheat and barley plant development, were completely different. The same function of *TaCKX10* is expected to be different from the known function of *HvCKX9*.

The starters for *TaCKX5* were found to hybridized to 3B and might be expressed only from this genome. The closest homologues of the investigated *TaCKX5* were *CKX5* orthologues belonging to several species: *S*. *cereale*, *S*. *brachyantha*, *S*. *bicolor*, *S*. *italica*, *B*. *distachyon* as well as *CKX5*-like *O*. *sativa*. None of them has been analysed. Nonetheless, *TaCKX5* seemed to be a very important element of the family, also showing the highest expression among the others in seedling roots and inflorescences and very high, just after *TaCKX1*, in 0 DAP spikes.

The primers for the third gene specific to leaves, *TaCKX4* [31], based on *T*. *aestivum* IWGSC assembly, hybridized to chromosome 3B and 3D. The gene was not homologous to *TaCKX4-1* to *4–3* copies characterized by Chang et al. [26], which were located to chromosome 3A. However, *TaCKX4* investigated by us was orthologous to *A*. *tauschii CKX4-like* with 94% identity, *H*. *vulgare CKX4* with 89% identity and *L*. *perenne CKX4* with 85% identity, suggesting that the number of the gene is correct. According to Song et al. [31], expression of *TaCKX4* was very low during reproductive development, which was in agreement with our data. The authors also found a low mRNA level in flag leaves, which differs from our results, probably because the developmental stage of well-developed leaves from young plants used by us was not comparable with the developmental stage and role of flag leaves. The only research on the role of *TaCKX4-1* to *4–3* was reported by Chang et al. [26], who merged copy number variation with grain weight and chlorophyll content in the RIL population. Unexpectedly, the two-copy locus of *TaCKX4* corresponded to higher chlorophyll content and grain weight compared to null or three-copy genotypes. These data are difficult to interpret, since we know nothing about expression of these copies of the *TaCKX4* gene. Higher copy number should correlate with higher expression and should increase CKX enzyme activity and irreversible cytokinin degradation. Since an increased level of CKs delays senescence and causes nutrient mobilization [33], which positively affects grain yield, one should expect that higher copy number should be negatively associated with chlorophyll content compared to the null phenotype. Anyway, *TaCKX4* investigated in this study was not comparable with three-copied *TaCKX4*, since the genes were not homologous and were located on different genomes.

The second group of *TaCKX* genes which were highly and specifically expressed in developing spikes were represented by *TaCKX1*,*TaCKX2*.*1*and *TaCKX2*.*2*. Amplicon hybridization with *T*. *aestivum* IWGSC assembly data showed that the *TaCKX1* was expressed from chromosomes 3A, 3B, 3D and both *TaCKX2*.*1* and *TaCKX2*.*2* from chromosomes 3B and 3D. Similar to our data, *TaCKX1* was the most highly expressed gene in developing spikes among the six tested by Song et al. [31] and the only one with high expression in mature and senescing flag leaves. Expression measured by semi-quantitative RT-PCR proved that the gene was not expressed in root, sheath, and leaf [4]. Phylogenetic analysis showed that *TaCKX1* shares high sequence similarity with other *CKX1*-type orthologues from *A*. *tauschii CKX1-like*, *S*. *cereale ScCKX1*, *H*. *vulgare CKX1*, and lower with *Z*. *mays CKX1*. Analysis of the transcript level of *HvCKX1* in different organs of developing barley plants showed a similar pattern of expression to wheat [12]. The level of transcript was the highest in 14 DAP spikes and was lower in 7 DAP and 0 DAP spikes. Stable RNAi silencing of *HvCKX1* expression was associated with decreased CKX enzyme activity in developing spikes leading to a higher number of seeds and higher plant productivity [22]. Taking into account the high similarity of *CKX1* orthologues, especially *HvCKX1*, which additionally showed close similarity to *TaCKX1* in wheat expression pattern in developing plants of barley, we might expect an important role of *TaCKX1* in common wheat productivity.

Former annotated five duplicates of *TaCKX2*: *TaCKX2*.*3*, *TaCKX2*.*2*, *TaCKX2*.*1*, *TaCKX2*.*4* and *TaCKX2*.*5* showed 2, 4, 8, 20 and 100 times lower expression respectively than in the case of *TaCKX1*. Profiles of expression of investigated by us genes are close to the result of *TaCKX2* and *TaCKX1* presented by Song et al. [31], however the levels of expression are not comparable since expression pattern for former five duplicates was not investigated. Semi-quantitative analysis proved high expression of two *TaCKX2* genes in young spikes and culms [6]. Moreover, there was a positive correlation between expression level of both genes and grain number per spike in 12 wheat varieties. As discussed for *TaCKX4*, we should expect a negative correlation between these traits. Phylogenetic analysis showed that *TaCKX2*.*2* is most closely related to *A*. *tauschii CKX2-like*, *H*. *vulgare CKX2*.*2* and *S*. *cereale ScCKX2*.*2* as well as sharing very high sequence similarity to *TaCKX2*.*2*(JN381555). Proteins encoded by *TaCKX2*.*2*(FJ648070), *TaCKX2*.*2*(GU084177) were found to be most closely related to *OsCKX2* and have been located in clustered clade I of monocots [6] and according to our results to *HvCKX2*.*2*. These data did not confirm the investigation by Mameaux et al. [3] showing that *TaCKX2* has five duplicated copies, since all three clones are located in one *TaCKX2*.*2* gene. The most similar sequence to *TaCKX2*.*2* found in databases was the *TaCKX6-D1b* allele isolated and analyzed by Zhang et al. [24]. Their expression patterns were similar, however differ from *TaCKX6* investigated in this research.

The *TaCKX9*, *TaCKX7*(JN128589) and *TaCKX7*(JN128588) expression was highly specific to seedling roots, but very low compared to other *TaCKX* in other organs tested including roots. Despite their very high correlation coefficient of expression (0.99–1.00), the genes were expressed from different chromosomes, 7A, 7B and 7D as well as 6B and 6D, respectively. Both, *TaCKX9* and *TaCKX7* were expressed from genome D, which was shown to play a crucial role in the increased lateral root number [34]. According to phylogenetic analysis the most closely related orthologues of *TaCKX7* was *H*. *vulgare CKX7* as well as protein encoded by the gene and for *TaCKX9 A*. *tauschii*, *CKX10-like*. None of these genes have been analysed.

*TaCKX3*, *TaCKX6* and *TaCKX11* were more or less expressed through all the organs tested. *TaCKX3* used in our research was isolated by Song et al. [31] and expression of the gene investigated by the same group was reported as very low during both reproductive stage and flag leaf development. The data are comparable with ours for developing spikes, but are not comparable in the case of leaves, which differ in stages of plant development. Because *TaCKX3* was expressed through the all organs tested, showing an average expression level among *TaCKX* genes, we set relative expression as 1.00 for the mean *TaCKX3* expression measured in all organs. The highest relative expression of the gene, ranging from 3 to 8, was revealed in well-developed leaves and was around 1.0 in 14 DAP and 0 DAP spikes. There are notably large differences between the high expression level of the gene in leaves of the old cultivar Kontesa and the modern cultivars Ostka and Trappe. Highly homologous and mapped to the same chromosome 7B, *TaCKX3* isolated by Ma et al. [7] had no signal peptide at the N terminus, which means that the gene functions in the cytoplasm. Very close orthologues of *TaCKX3* were *A*. *tauschii CKX11*, *S*. *cereale CKX11* and one clone of *H*. *vulgare*. 97% similarity was shown by *O*. *sativa CKX11-like* and *Z*. *mays CKX10*. Despite close similarity of *TaCKX3* to *CKX11* orthologues, *TaCKX11* showed a different pattern of expression and chromosome localisation compared to *TaCKX3*. The closest orthologue to *TaCKX11* was *H*. *vulgare CKX8*, sharing 95% similarity as well as protein encoded by the gene; less close was orthologue of *Z*. *mays CKX12*.

*TaCKX6* investigated by us, isolated and shown as the third highly expressed *TaCKX* in developing spikes by Song et al. [31], in our case was the seventh one taking into account the level of expression, and showed higher expression in leaf, 14 DAP spikes and seedling roots than in other organs tested. These results are not comparable because of using different objects/cultivars, organs, reference genes, number of tested *TaCKX* genes and the way of counting results. The coding sequence of *TaCKX6* used by us was located on chromosomes 1A, 1B and 1D, but it was not similar to *TaCKX6-D1b* or -*D1a* isolated and characterized by Zhang et al. [24]. Haplotype *TaCKX6-D1a* has an 18 nt deletion compared to *TaCKX6-D1b*, and its expression was negatively associated with higher grain weight. Performed by us transcript analysis revealed that *TaCKX6-D1* is indeed the *TaCKX2*.*2* and is located on chromosome 3. Another *TaCKX6* allele associated with grain size, filling rate and weight, *TaCKX6a02* [25] was located on chromosome 3DS, but again was not similar to the one investigated by us or *TaCKX6-D1*. Performed by us transcript analysis revealed that *TaCKX66a02* is in fact the *TaCKX2*.*1* which was located on chromosome 3D. Results of alignment proved that all three *TaCKX6* genes differed in their close relationship with other *TaCKX* genes and their orthologues. The *TaCKX6* located on 1D was the closest homologue of *T*. *aestivum CKX8*, sharing 98% identity under 89% cover of the sequences. The closest orthologue was *H*. *vulgare CKX3* with 94% identity and *L*. *perenne CKX6* with 96% identity. *Z*. *mays CKX6* shared 81% identity under 83% coverage. The *TaCKX-D1* located on 3D was the closest homologue of *TaCKX2*.*2*, sharing 98% identity, and *TaCKX2*.*1* with 97% identity, and under 43% cover for both. Their close orthologues were *A*. *tauschii CKX2-like* with 99% identity and 43% cover and *H*. *vulgare CKX2*.*2* (69% cover/88% identity). The closest homologue of *TaCKX6a02* located on 3D was *T*. *aestivum CKX2*.*1*(JF293079) with 99% identity under 100% cover. Summarizing alignment and phylogenetic tree analysis, *TaCKX6* investigated by us was among *TaCKX3*, *5*, *4*, *8*, *9* and the closest to other *CKX6* orthologues. Since *TaCKX6* is not the same as *TaCKX6-D1* characterized by Zhang et al. [24] as well as *TaCKX6-D1a* by Lu et al. [25], the genes probably would not share the same function.

### Developmental and daily time dependence of *TaCKX* expression levels and their co-operation

The *TaCKX1*, *TaCKX2*.*2* and *TaCKX2*.*1* genes, which are specific to developing spikes, and unspecific but well expressed in this organ *TaCKX3* were shown to be developmentally and daily time dependent. The level of expression of *TaCKX1* and *TaCKX3* was significantly higher in the first 7 DAP spikes comparing to the second 7 DAP spikes in two or three cultivars and the result was opposed to *TaCKX2*.*1*. Similar data were observed for daily time expression of *TaCKX1*, *TaCKX2*.*1* and *TaCKX3* in 7 DAP spikes, which was highest at 9:00 am, lower at 3:00 pm and lowest at 12:00 pm and various for *TaCKX2*.*1*. These differences in developmental and daily time expression of *TaCKX2*.*1* and *TaCKX2*.*2* as well as their different levels of expression indicate that detailed functions of the *TaCKX2* genes might be different. The data of respectively high and specific expression of *TaCKX1*, *TaCKX2*.*1* and *TaCKX2*.*2* genes were compatible with their strong correlation coefficients of expression in investigated organs assuming their powerful cooperation in spike development. Daily time dependence of expression in 7 DAP spikes was also significant for *TaCKX3* and low expressing genes. These data showed that developmental and daily time expression of most *TaCKX* genes was cultivar independent, but some of them are developmentally and day-time insensitive.

Besides the very high correlation coefficients of expression in the group of spike-specific genes the correlation was also very high among leaf-specific genes: *TaCKX4*, *TaCKX5*, *TaCKX10* and highly expressed in this organ *TaCKX3*. Generally we showed that there were two groups of *TaCKX* genes which positively cooperate in developing wheat plants. The first groups genes expressed in organs from young plants: seedling roots, leaves from 4-week-old plants, inflorescences and 0 DAP spikes. The second group includes *TaCKX* genes expressed in developing spikes of maturing plants: 0 DAP, 7 DAP and 14 DAP, reaching a very high correlation coefficient.

### Differential expression of the genotypes

Although the expression patterns of individual *TaCKX* genes are tissue- and developmentally-specific, their expression levels measured in individual organs in most cases significantly differ among three cultivars used in the experiments. These differences are more distinct in the larger group of breeding material (not published yet) and might be result of various alleles of the tested genes, indicating for possible selection of these alleles of interest for breeding.

### Looking for *TaCKX* genes regulating yield-related traits

The goal of this research was to select those *TaCKX* genes which might regulate yield-related traits in wheat. To achieve the goal we hypothesized that expression patterns of *TaCKX* genes indicate their role in growth and reproductive development. The same hypothesis for *HvCKX* genes has been positively verified for barley [12]. The data with barley showed that RNAi silencing of *HvCKX* genes that are highly and specifically expressed in developing spikes led to higher productivity [22, 23]. This effect was the consequence of a decreased level of expression of selected *HvCKX* genes, which was associated with a decrease of CKX enzyme activity. Since CKX enzyme irreversibly degrades cytokinins, a decreased level of expression of the *TaCKX* genes in selected organs is expected to increase cytokinin content. High levels of CKs promote numerous developmental features, which in the case of rice [21] and barley spikes [22, 23] led to higher grain numbers. Assuming this, yield-related traits in wheat might be regulated most of all by those *TaCKX* genes which are specifically expressed in developing spikes. We found three of 11 newly numbered, *TaCKX1*, *TaCKX2*.*2* and *TaCKX2*.*1*, highly specific to inflorescences and developing spikes and a fourth, *TaCKX3*, which is not specific but is relatively highly expressed in the same organs. As discussed above, a positive effect of these genes on yield-related traits might operate on similar mechanism as in barley or rice. We chose two ways to reach this goal and positively verify the hypothesis. The first is to decrease selected gene expression by direct silencing, as was done in barley or rice. The second one is to look for natural variation of expression of these genes in generative organs among wheat genotypes. Preliminary, not yet published results prove that both ways might lead to the goal. Moreover, the first way provides us well-characterized material for detailed, functional analysis of the silenced genes. The second way gives us an opportunity to select non-GMO breeding lines for breeding purposes.

## Supporting information

S1 Table Primer sequences designed for reference gene *Ref2* and each *TaCKX* gene. (PDF)

S2 Table Homologous sequences of *TaCKX* and *HvCKX* retrieved from Ensembl Plants database. (PDF)

S1 File Figs A-N. The distance tree of pairwise comparison of *TaCKX1* (A), *TaCKX2.1* (B), *TaCKX2.2* (C), *TaCKX3* (D), *TaCKX4* (E), *TaCKX5* (F), *TaCKX6* (G), *TaCKX6-D1* (H), *TaCKX6a02* (I), *TaCKX7* (J), *TaCKX8* (K), *TaCKX9* (L), *TaCKX10* (M), *TaCKX11* (N) with their homologues and orthologues (queries are highlighted by yellow).
(PDF)

S1 Fig Phylogenic three of TaCKX and HvCKX amino acid sequences. (JPG)

## References

[1] Curtis T, Halford NG. Food security: the challenge of increasing wheat yield and the importance of not compromising food safety. Ann Appl Biol. 2014;164(3):354–72. 10.1111/aab.12108 WOS:000334359200005. 25540461 PMC4240735

[2] Nadolska-Orczyk A, Rajchel IK, Orczyk W, Gasparis S. Major genes determining yield-related traits in wheat and barley. Theor Appl Genet. 2017;130(6):1081–98. 10.1007/s00122-017-2880-x WOS:000401980500001. 28314933 PMC5440550

[3] Mameaux S, Cockram J, Thiel T, Steuernagel B, Stein N, Taudien S, et al Molecular, phylogenetic and comparative genomic analysis of the *cytokinin oxidase/dehydrogenase* gene family in the *Poaceae*. Plant Biotechnol J. 2012;10(1):67–82. 10.1111/j.1467-7652.2011.00645.x WOS:000298094800008. 21838715

[4] Feng DS, Wang HG, Zhang XS, Kong LR, Tian JC, Li XF. Using an Inverse PCR Method to Clone the Wheat *Cytokinin Oxidase/Dehydrogenase Gene TaCKX1*. Plant Mol Biol Rep. 2008;26(3):143–55. 10.1007/s11105-008-0033-8 WOS:000259962600001.

[5] Lei Z, Baoshi Z, Ronghua Z. Isolation and chromosomal localization of cytokinin oxidase/dehydrogenase gene (*TaCKX5*) in wheat. Scientia Agricultura Sinica 2008;41:636–642.

[6] Zhang JP, Liu WH, Yang XM, Gao AN, Li XQ, Wu XY, Li LH. Isolation and characterization of two putative *cytokinin oxidase* genes related to grain number per spike phenotype in wheat. Mol Biol Rep. 2011;38(4):2337–47. 10.1007/s11033-010-0367-9 WOS:000289257100017. 21104150

[7] Ma X, Feng DS, Wang HG, Li XF, Kong LR. Cloning and Expression Analysis of Wheat *Cytokinin Oxidase/Dehydrogenase Gene TaCKX3*. Plant Mol Biol Rep. 2011;29(1):98–105. 10.1007/s11105-010-0209-x WOS:000286674600011.

[8] Hare PD, Vanstaden J. Cytokinin oxidase—Biochemical Features and Physiological Significance. Physiol Plantarum. 1994;91(1):128–36. 10.1034/j.1399-3054.1994.910118.x WOS:A1994NN55600018.

[9] Jones RJ, Schreiber BMN. Role and function of cytokinin oxidase in plants. Plant Growth Regul. 1997;23(1–2):123–34. 10.1023/A:1005913311266 WOS:000071317800008.

[10] Mok DWS, Mok MC. Cytokinin metabolism and action. Annu Rev Plant Phys. 2001;52:89–118. 10.1146/annurev.arplant.52.1.89 WOS:000169615600005. 11337393

[11] Werner T, Schmulling T. Cytokinin action in plant development. Curr Opin Plant Biol. 2009;12(5):527–38. 10.1016/j.pbi.2009.07.002 WOS:000271137200003. 19740698

[12] Zalewski W, Gasparis S, Boczkowska M, Rajchel IK, Kala M, Orczyk W, et al Expression patterns of *HvCKX* genes indicate their role in growth and reproductive development of barley. Plos One. 2014;9(12):e115729 10.1371/journal.pone.0115729 25531889 PMC4274103

[13] Song J, Jiang L, Jameson PE. Identification and quantitative expression of cytokinin regulatory genes during seed and leaf development in wheat. Agronomy Society of New Zealand Special Publication No l3 / Grassland Research and Practice Series No 14. 2015.

[14] Kudo T, Kiba T, Sakakibara H. Metabolism and Long-distance Translocation of Cytokinins. J Integr Plant Biol. 2010;52(1):53–60. 10.1111/j.1744-7909.2010.00898.x WOS:000274795500007. 20074140

[15] Kuiper D. Sink Strength—Established and Regulated by Plant-Growth Regulators. Plant Cell Environ. 1993;16(9):1025–6. 10.1111/j.1365-3040.1996.tb02052.x WOS:A1993MH66800006.

[16] Roitsch T, Ehness R. Regulation of source/sink relations by cytokinins. Plant Growth Regul. 2000;32(2–3):359–67. 10.1023/A:1010781500705 WOS:000167858500031.

[17] Werner T, Holst K, Pors Y, Guivarc'h A, Mustroph A, Chriqui D, et al Cytokinin deficiency causes distinct changes of sink and source parameters in tobacco shoots and roots. J Exp Bot. 2008;59(10):2659–72. 10.1093/jxb/ern134 WOS:000257401500008. 18515826 PMC2486470

[18] Schmulling T, Werner T, Riefler M, Krupkova E, Bartrina y Manns I. Structure and function of *cytokinin oxidase*/*dehydrogenase* genes of maize, rice, *Arabidopsis* and other species. J Plant Res. 2003;116(3):241–52. 10.1007/s10265-003-0096-4 WOS:000183664100009. 12721786

[19] Werner T, Motyka V, Strnad M, Schmulling T. Regulation of plant growth by cytokinin. P Natl Acad Sci USA. 2001;98(18):10487–92. 10.1073/pnas.171304098 WOS:000170738000083. 11504909 PMC56987

[20] Werner T, Kollmer I, Bartrina I, Holst K, Schmulling T. New insights into the biology of cytokinin degradation. Plant Biology. 2006;8(3):371–81. 10.1055/s-2006-923928 WOS:000238359400013. 16807830

[21] Ashikari M, Sakakibara H, Lin SY, Yamamoto T, Takashi T, Nishimura A, et al Cytokinin oxidase regulates rice grain production. Science. 2005;309(5735):741–5. 10.1126/science.1113373 WOS:000230938200041. 15976269

[22] Zalewski W, Galuszka P, Gasparis S, Orczyk W, Nadolska-Orczyk A. Silencing of the *HvCKX1* gene decreases the cytokinin oxidase/dehydrogenase level in barley and leads to higher plant productivity. J Exp Bot. 2010;61(6):1839–51. 10.1093/jxb/erq052 WOS:000276735300024. 20335409

[23] Zalewski W, Orczyk W, Gasparis S, Nadolska-Orczyk A. *HvCKX2* gene silencing by biolistic or *Agrobacterium*-mediated transformation in barley leads to different phenotypes. Bmc Plant Biol. 2012;12:206 10.1186/1471-2229-12-206 23134638 PMC3541248

[24] Zhang L, Zhao YL, Gao LF, Zhao GY, Zhou RH, Zhang BS, et al *TaCKX6*-D1, the ortholog of rice *OsCKX2*, is associated with grain weight in hexaploid wheat. New Phytol. 2012;195(3):574–84. 10.1111/j.1469-8137.2012.04194.x WOS:000306179200011. 22670578

[25] Lu J, Chang C, Zhang HP, Wang SX, Sun G, Xiao SH, et al Identification of a Novel Allele of *TaCKX6a02* Associated with Grain Size, Filling Rate and Weight of Common Wheat. Plos One. 2015;10(12):e0144765 10.1371/journal.pone.0144765 26657796 PMC4685998

[26] Chang C, Lu J, Zhang HP, Ma CX, Sun GL. Copy Number Variation of *Cytokinin Oxidase Gene Tackx4* Associated with Grain Weight and Chlorophyll Content of Flag Leaf in Common Wheat. Plos One. 2015;10(12):15 10.1371/journal.pone.0145970 WOS:000367481900107. 26714276 PMC4699907

[27] Kersey PJ, Allen JE, Allot A, Barba M, Boddu S, Bolt BJ, et al Ensembl Genomes 2018: an integrated omics infrastructure for non-vertebrate species. Nucleic Acids Res. 2018;46(D1):D802–D8. 10.1093/nar/gkx1011 WOS:000419550700119. 29092050 PMC5753204

[28] Katoh K, Standley DM. 2013 MAFFT Multiple Sequence Alignment Software Version 7: Improvements in Performance and Usability. Molecular Biology and Evolution 30, 772–780. 10.1093/molbev/mst010 23329690 PMC3603318

[29] Katoh K, Rozewicki J, Yamada KD. 2017 MAFFT online service: multiple sequence alignment, interactive sequence choice and visualization. Brief Bioinform. 10.1093/bib/bbx108 PMC6781576 28968734

[30] Kumar S, Stecher G, Li M, Knyaz C, Tamura K. 2018 MEGA X: Molecular Evolutionary Genetics Analysis across Computing Platforms. Molecular Biology and Evolution 35, 1547–1549. 10.1093/molbev/msy096 29722887 PMC5967553

[31] Song J, Jiang L, Jameson PE. Co-ordinate regulation of cytokinin gene family members during flag leaf and reproductive development in wheat. Bmc Plant Biol. 2012;12:78 10.1186/1471-2229-12-78 22672647 PMC3410795

[32] Mrizova K, Jiskrova E, Vyroubalova S, Novak O, Ohnoutkova L, Pospisilova H, et al Overexpression of *cytokinin dehydrogenase* genes in barley (*Hordeum vulgare* cv. Golden Promise) fundamentally affects morphology and fertility. Plos One. 2013;8(11):e79029 10.1371/journal.pone.0079029 24260147 PMC3829838

[33] Lara MEB, Garcia MCG, Fatima T, Ehness R, Lee TK, Proels R, et al Extracellular invertase is an essential component of cytokinin-mediated delay of senescence. Plant Cell. 2004;16(5):1276–87. 10.1105/tpc.018929 WOS:000221461400016. 15100396 PMC423215

[34] Wang HF, Hu ZR, Huang K, Han Y, Zhao AJ, Han HM, et al Three genomes differentially contribute to the seedling lateral root number in allohexaploid wheat: evidence from phenotype evolution and gene expression. Plant J. 2018;95(6):976–87. 10.1111/tpj.14005 WOS:000443813300005. 29932270

