# Supplementary material for: Specificity of expression of TaCKX family genes in developing plants of wheat and their co-operation within and among organs
Source: PLoS One. 2019 Apr 10;14(4):e0214239. doi: 10.1371/journal.pone.0214239 (PMC6457499; doi:10.1371/journal.pone.0214239)
Supplement: S1 Table — (PDF) [file pone.0214239.s001.pdf]

Table S1. Primer sequences designed for reference gene *Ref2* and each of *TaCKX* genes

Ref 2	Ta2291R	NCBI	GCTTCTGCCTGTCACATACGC	165
	Ta2291F		GCTCTCCAACAACATTGCCAAC	
TaCKX1	TaCKX1_188R		CCCAGGTACTCCTTGTACCCTAT	188
	TaCKX1_188F		GTCTACCCGCTCAACAAATCC	
TaCKX2.1	TaCKX2_1_R_205		TATCACATACGCCATCCATGC	205
	TaCKX2_1_F_205		TTGATCGCGGAGCTAATCCA	
TaCKX2.2	TaCKX2_2_R_175		ATCGTATCCTGGCCTCCTCA	175
	TaCKX2_2_F_175		TACCCCATGAACCGGAACAG	
TaCKX2.3	TaCKX2_3_R_144		TCTCCTCGTTCTGCTCCTCC	144
	TaCKX2_3_F_144		TCTACCCCATGAACCGGGAC	
TaCKX2.4	TaCKX2_4_R_220		GCCAAAAGTCACGATCCCCT	220
	TaCKX2_4_F_220		CTTCGTCCTTCTTCTCGGGC	
TaCKX2.5	TaCKX2_5_147R		GGGCAGACAAAGCTAGACCA	147
	TaCKX2_5_147F		GCTAGCCAGAGCTACACGG	
TaCKX3	TaCKX3_150R		GAATTAGAGTTCACGGCTTGATG	150
	TaCKX3_150F		TTGTCAAGGGACTGTAGTAGGG	
TaCKX4	TaCKX4_112_R		CTCCAAAGTCACACCCTCTACAC	112
	TaCKX4_112_F		AAGAACACGCAGCATAGCAAC	
TaCKX5	TaCKX5_3B_4R		CATACATGACACCAACGTACATCTT	150
	TaCKX5_3B_4F		GTCCGATTTTTGAGAAGACTGATT	
TaCKX6	TaCKX6_182R		CCGTGCTTGAATGTCTGC	182
	TaCKX6_182F		CACAAAGGAGGAGAAGGAGATG	
TaCKX7	TaCKX7_R_144		CTCGACTGTGGGTGGCTTC	144
	TaCKX7_F_144		CGGTCACGCATTCTCGACTT	
TaCKX8	TaCKX8_R_198		GGCGGTCGTCTCATCATAGT	198
	TaCKX8_F_198		TCTAAGCAGGCTAGCGAAGC	
TaCKX9	TaCKX9_R_278		GTCCCTGTTCATGGGGTACA	278
	TaCKX9_F_278		CCACGGTGGATCAGAAGCTC	
TaCKX10	TaCKX10_R_167		ACATAAAGCAATTTACCTGGACTTG	167
	TaCKX10_F_167		GAGCTAAGGGCTTGTGGGA	
TaCKX11	TaCKX11_R_184		AGTCATGCACTGCAAACTCTATG	184
	TaCKX11_F_184		GACCAAGAGCTTCTGATCTCAAT	
