# Supplementary material for: Specificity of expression of TaCKX family genes in developing plants of wheat and their co-operation within and among organs
Source: PLoS One. 2019 Apr 10;14(4):e0214239. doi: 10.1371/journal.pone.0214239 (PMC6457499; doi:10.1371/journal.pone.0214239)
Supplement: S2 Table — (PDF) [file pone.0214239.s002.pdf]

S2 Table. Homologous sequences of *TaCKX* and *HvCKX* retrieved from Ensemble Plants database.

Gene	Accesion numer NCBI	Accesion numer EP	Chrom. location	Transcript	No. of exons/ coding exons	Protein (aa)	Ref.
HvCKX1	JF495479,AF362472, BQ462284	HORVU3Hr1G019850	3H	HORVU3Hr1G019850.1	3	521	[3]
HvCKX2.1	JF495488, JF293078, JF293074	HORVU3Hr1G027460	3H	HORVU3Hr1G027460.1	3	598	[3]
				HORVU3Hr1G027460.2	4	561	
HvCKX2.2	JF495489, JF293077, JF293076, JF293075	HORVU3Hr1G027430	3H	HORVU3Hr1G027430.1	2	264	[3]
HvCKX3	JF495480	HORVU1Hr1G042360	1H	HORVU1Hr1G042360.1	5	522	[3]
				HORVU1Hr1G042360.2	6/5	522	
				HORVU1Hr1G042360.8	6	528	
HvCKX4	JF495481, BJ479455, BJ479606	HORVU3Hr1G105360	3H	HORVU1Hr1G042360.1	5	530	[3]
HvCKX5	JF495482, AK370106, BF264028, CB877904	HORVU3Hr1G075920	3H	HORVU3Hr1G075920.1	5	532	[3]
HvCKX7	JF495483	HORVU6Hr1G039690	6H	HORVU6Hr1G039690.1	1	533	[3]
		HORVU6Hr1G039680	6H	HORVU6Hr1G039680.1	1	533	
HvCKX8	AJ234763, JF495487	HORVU2Hr1G090150	2H	HORVU2Hr1G090150.3	5	292	[3]
		HORVU2Hr1G090160	2H	HORVU2Hr1G090160.5	5	334	
HvCKX9 (former *HxCKX2*)	AF540382, AV835311, AF490591, AY209184, JF495484	HORVU1Hr1G057860	1H	HORVU1Hr1G057860.6	5	526	[3,12]
				HORVU1Hr1G057860.10	5	526	
HvCKX10	JF495485	HORVU7Hr1G086710	7H	nontranslating CDS			[3]
HvCKX11	JF495486, CA031729	HORVU7Hr1G118130	7H	HORVU7Hr1G118130.3	6	515	[3]
				HORVU7Hr1G118130.4	6	511	
				HORVU7Hr1G118130.5	6	503	
