# Supplementary figures and images for: Specificity of expression of TaCKX family genes in developing plants of wheat and their co-operation within and among organs
Source: PLoS One. 2019 Apr 10;14(4):e0214239. doi: 10.1371/journal.pone.0214239 (PMC6457499; doi:10.1371/journal.pone.0214239)

**A**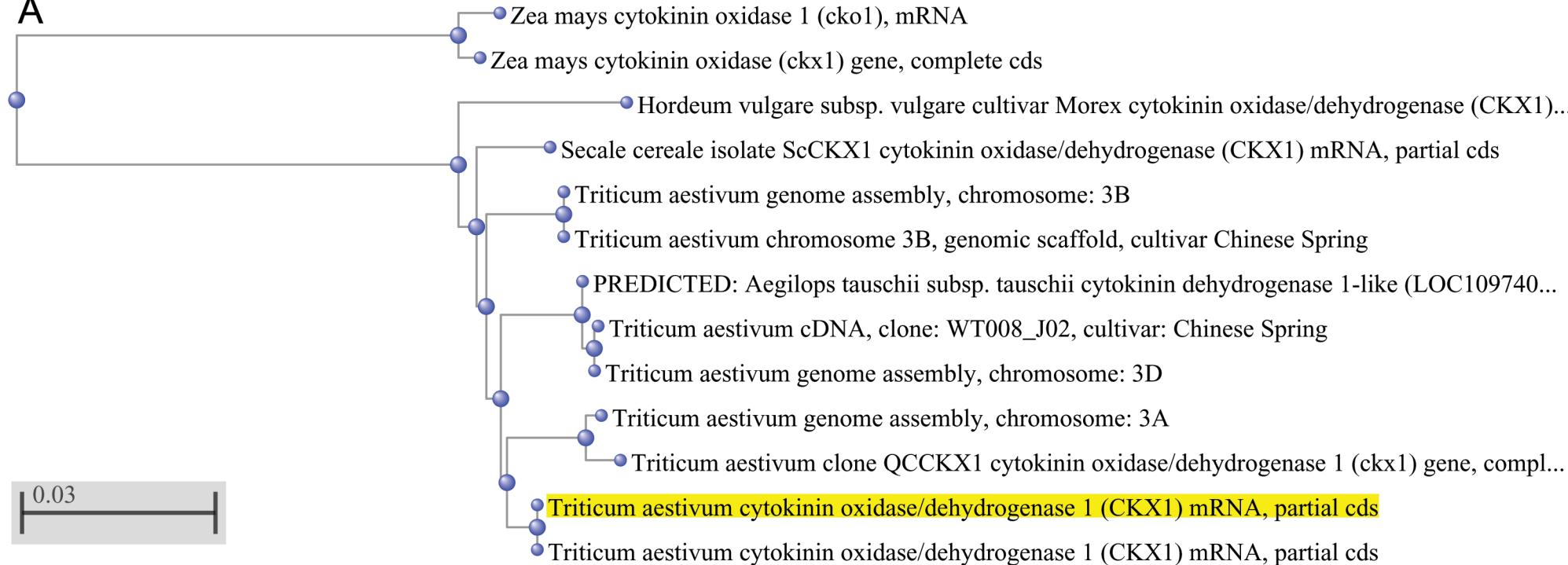

**B**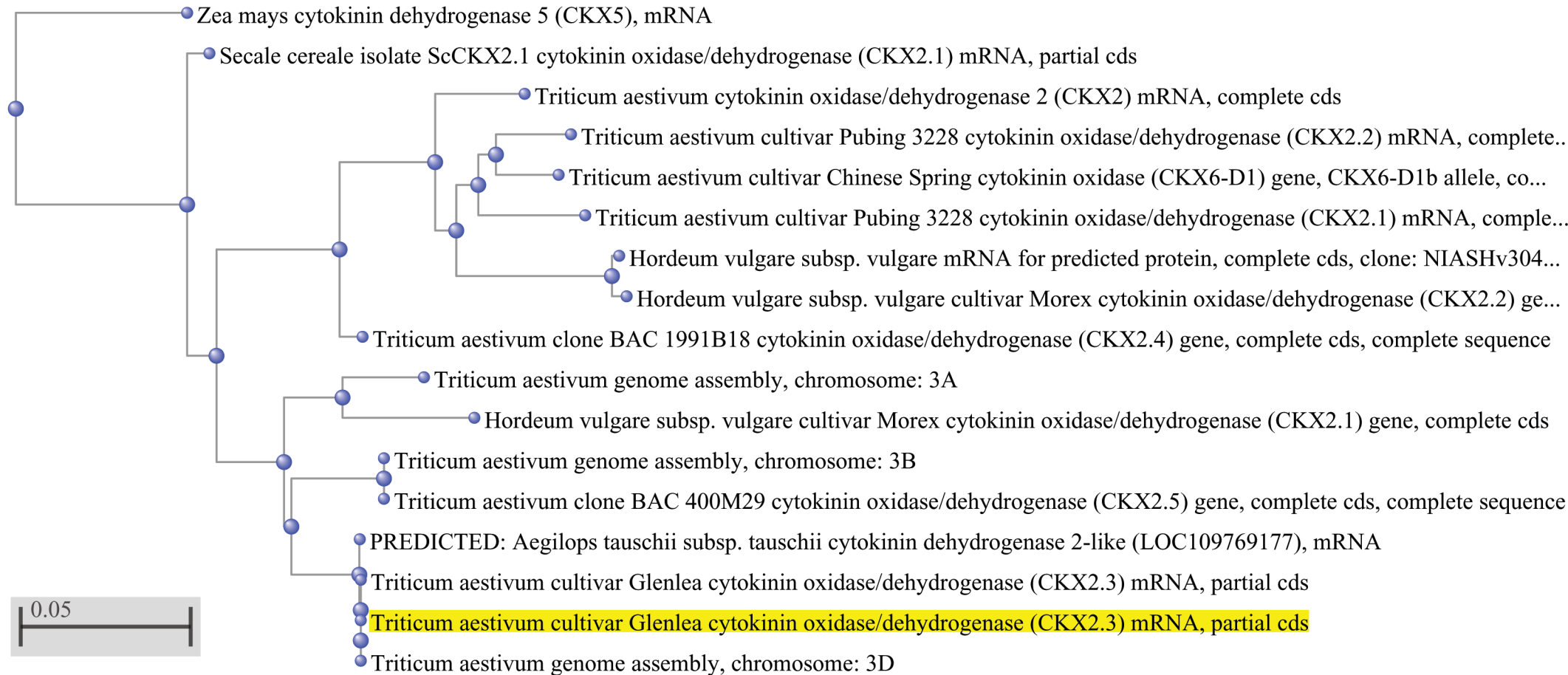

C

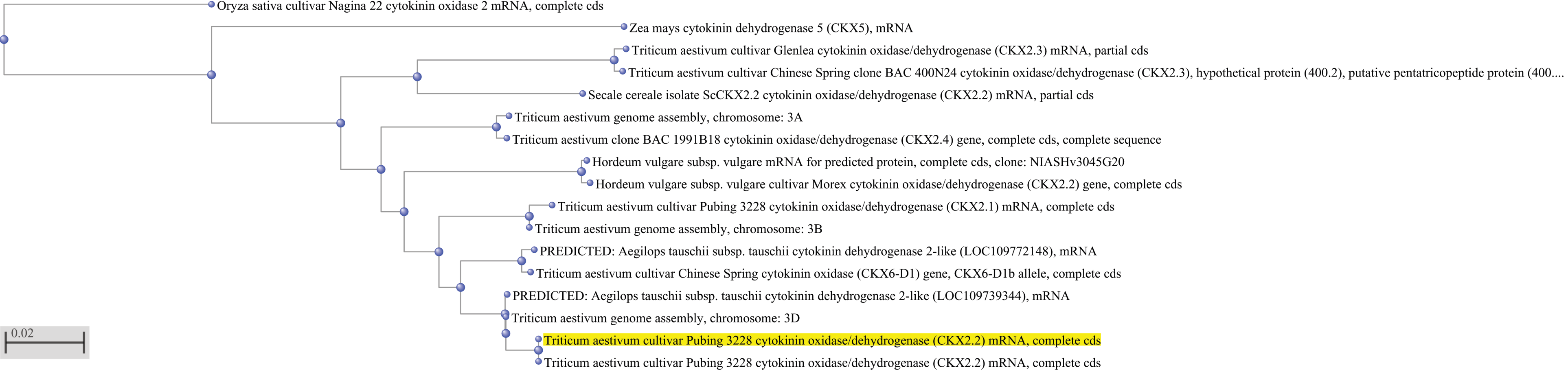

D

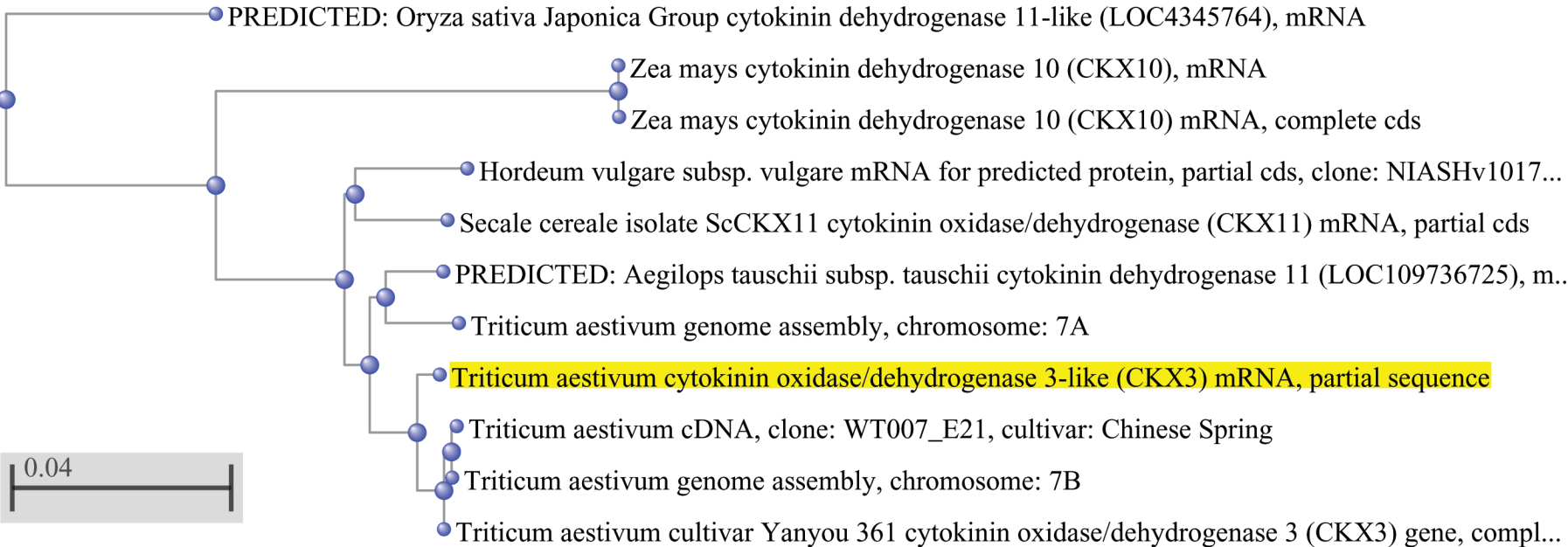

E

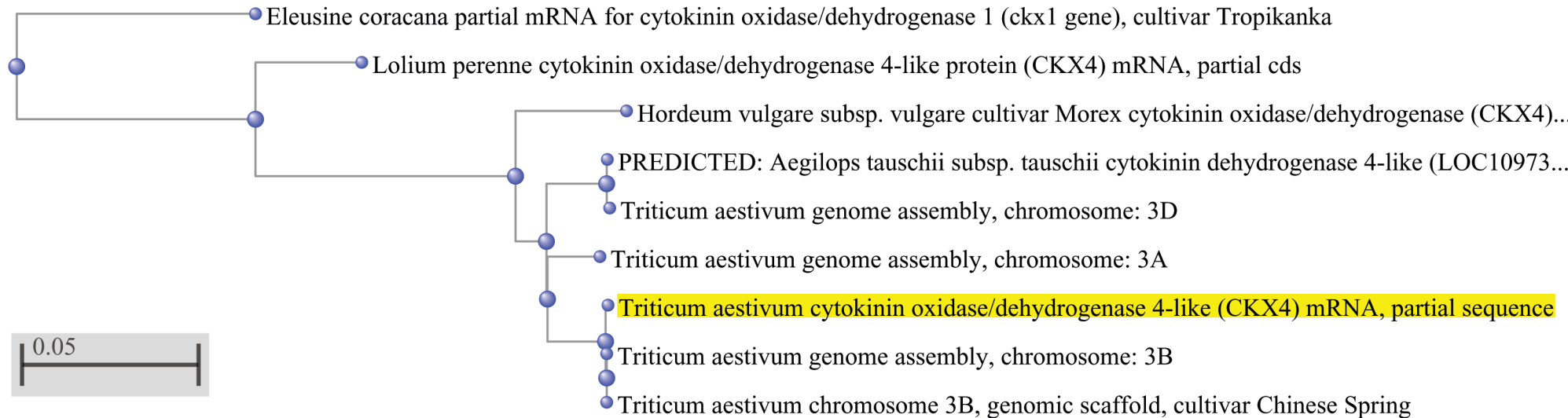

F

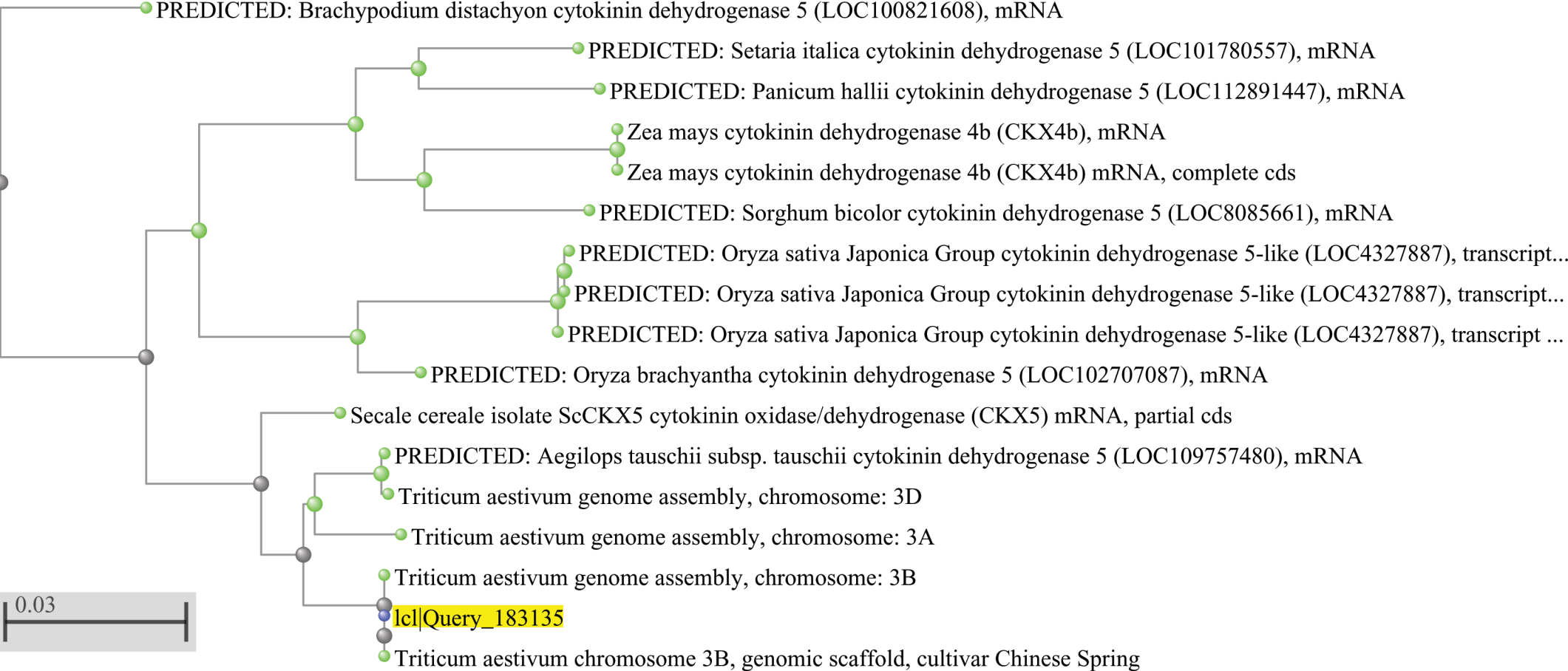

G

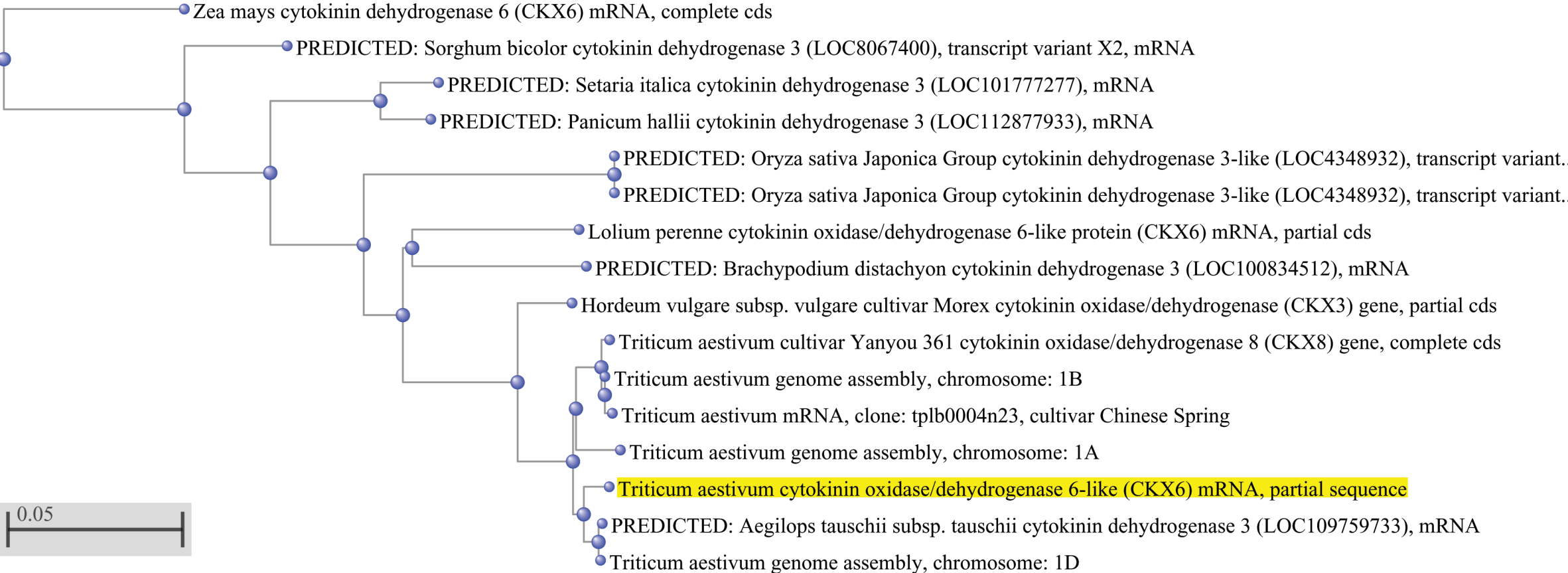

H

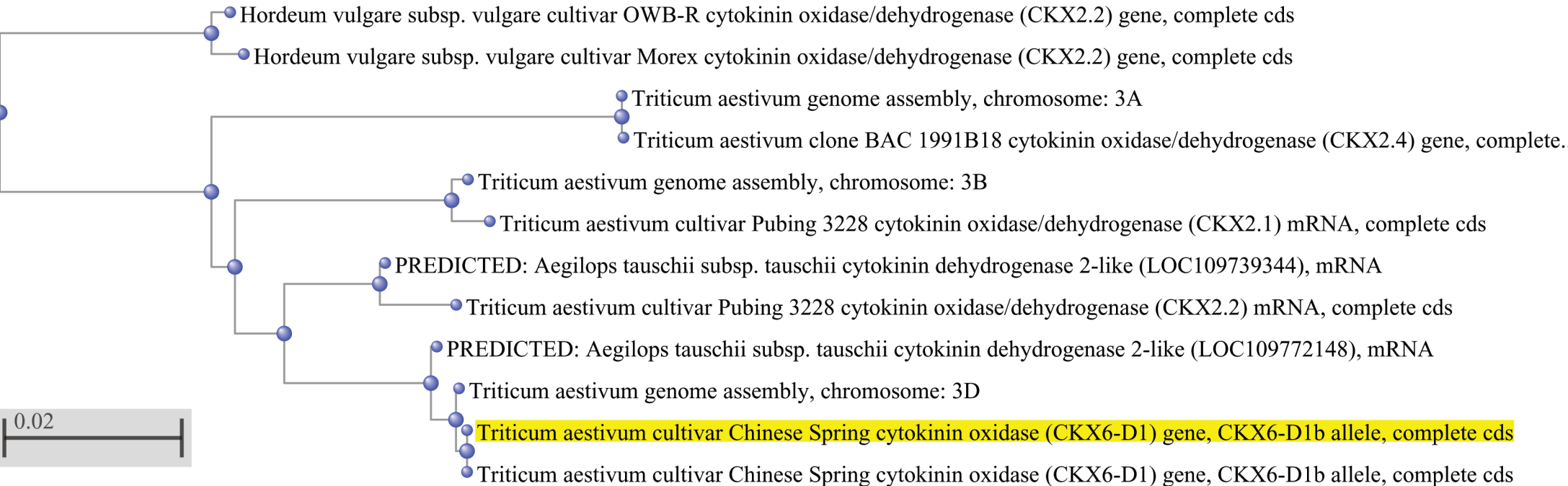

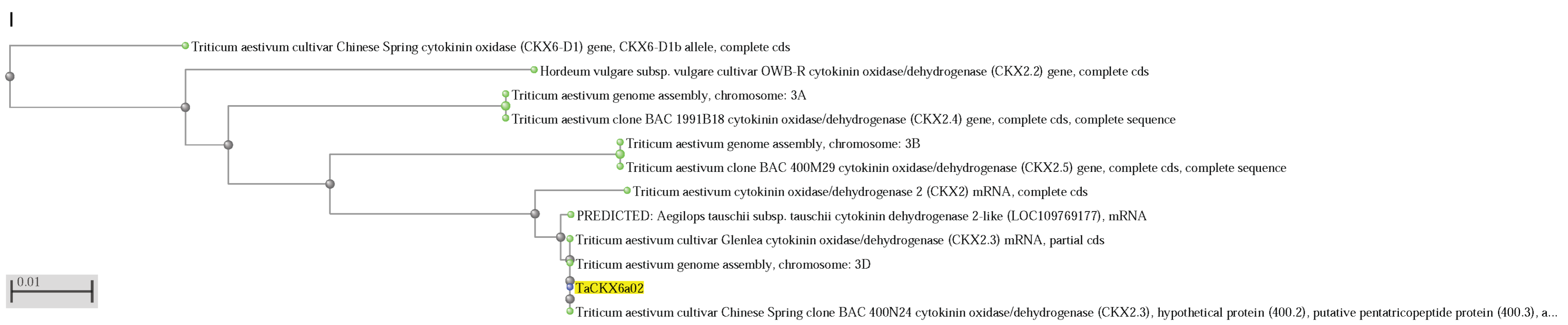

J

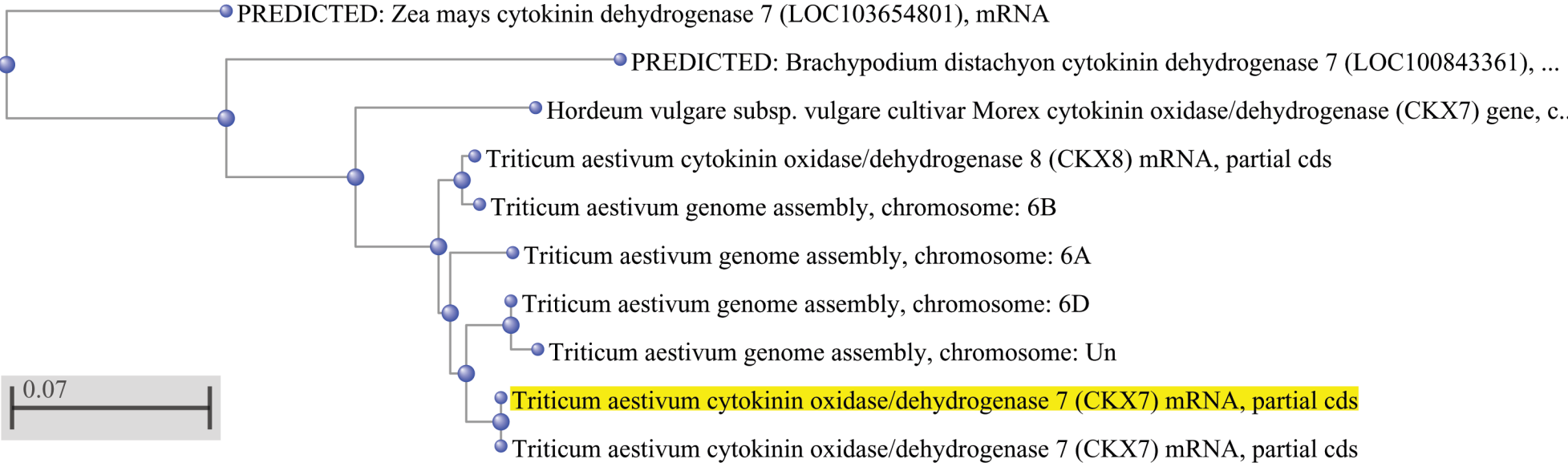

K

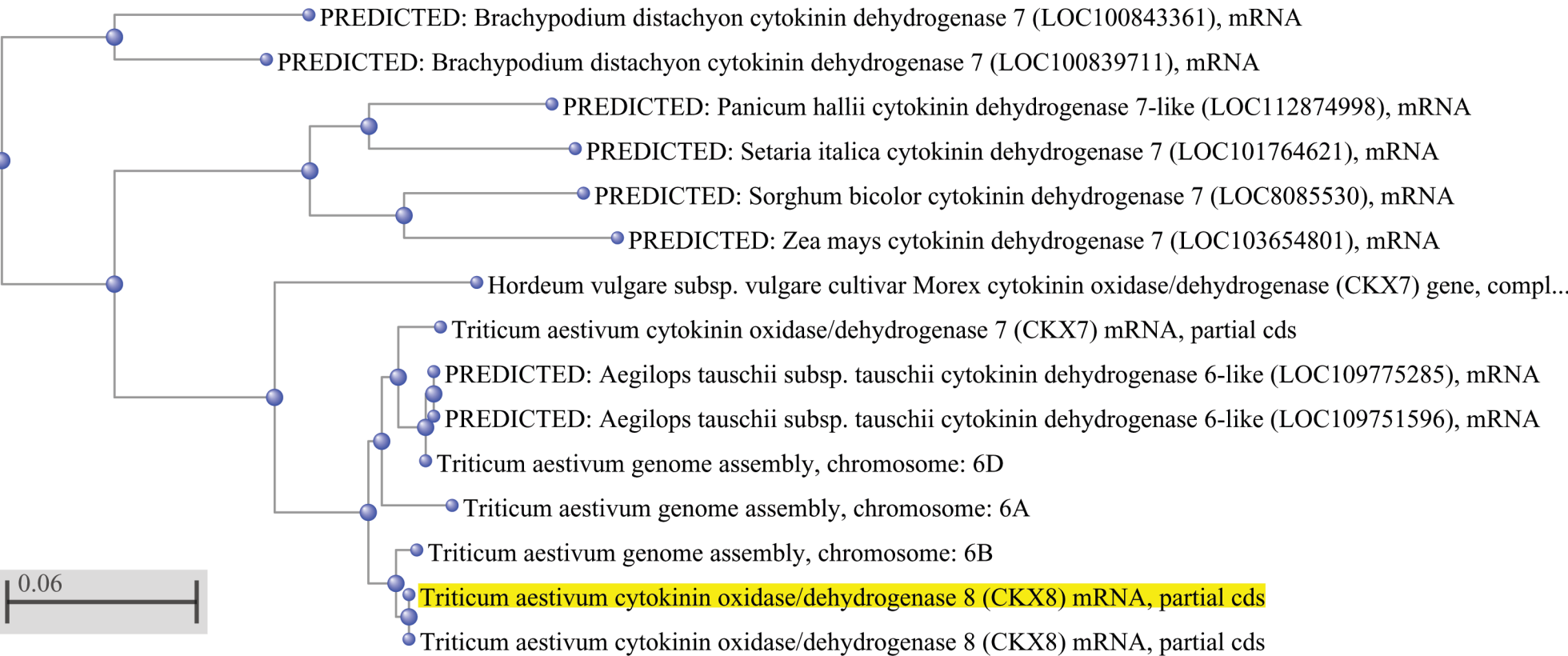

L

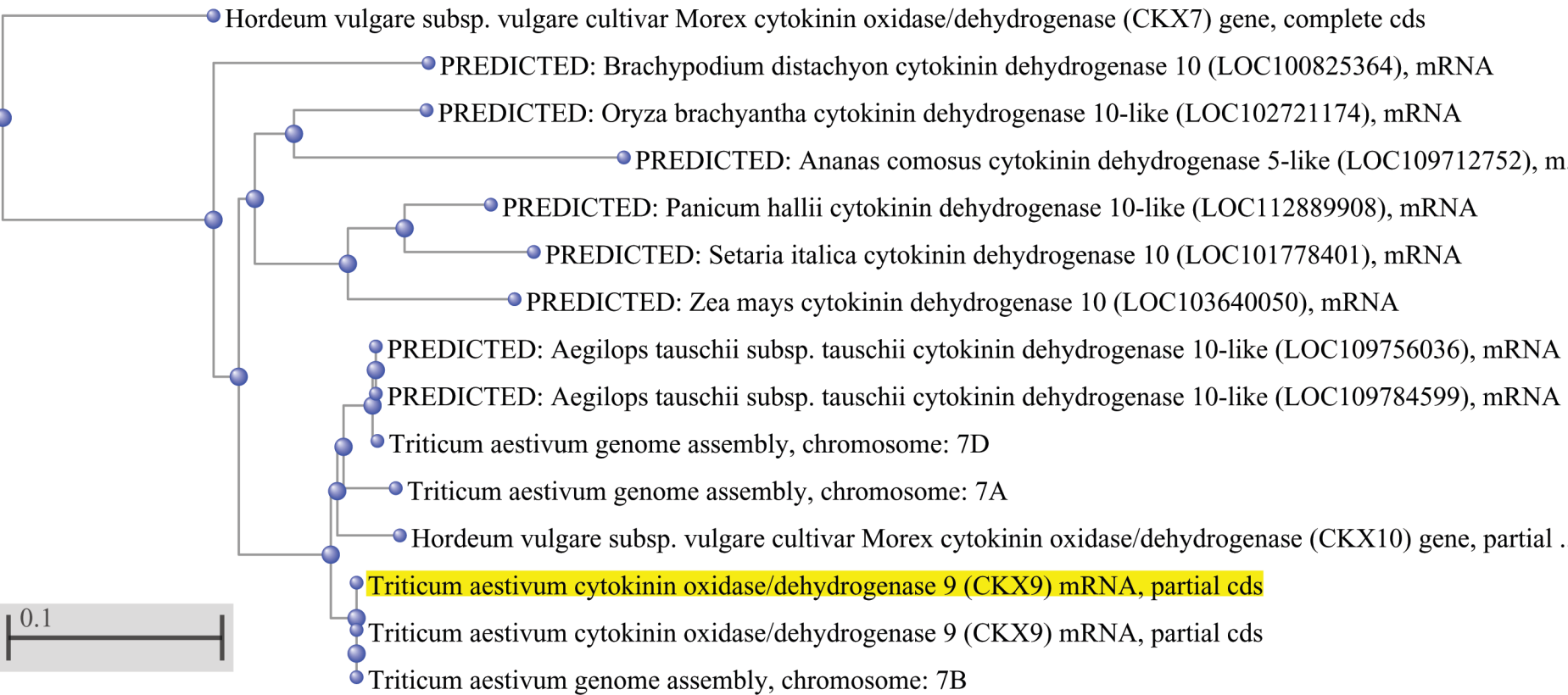

M

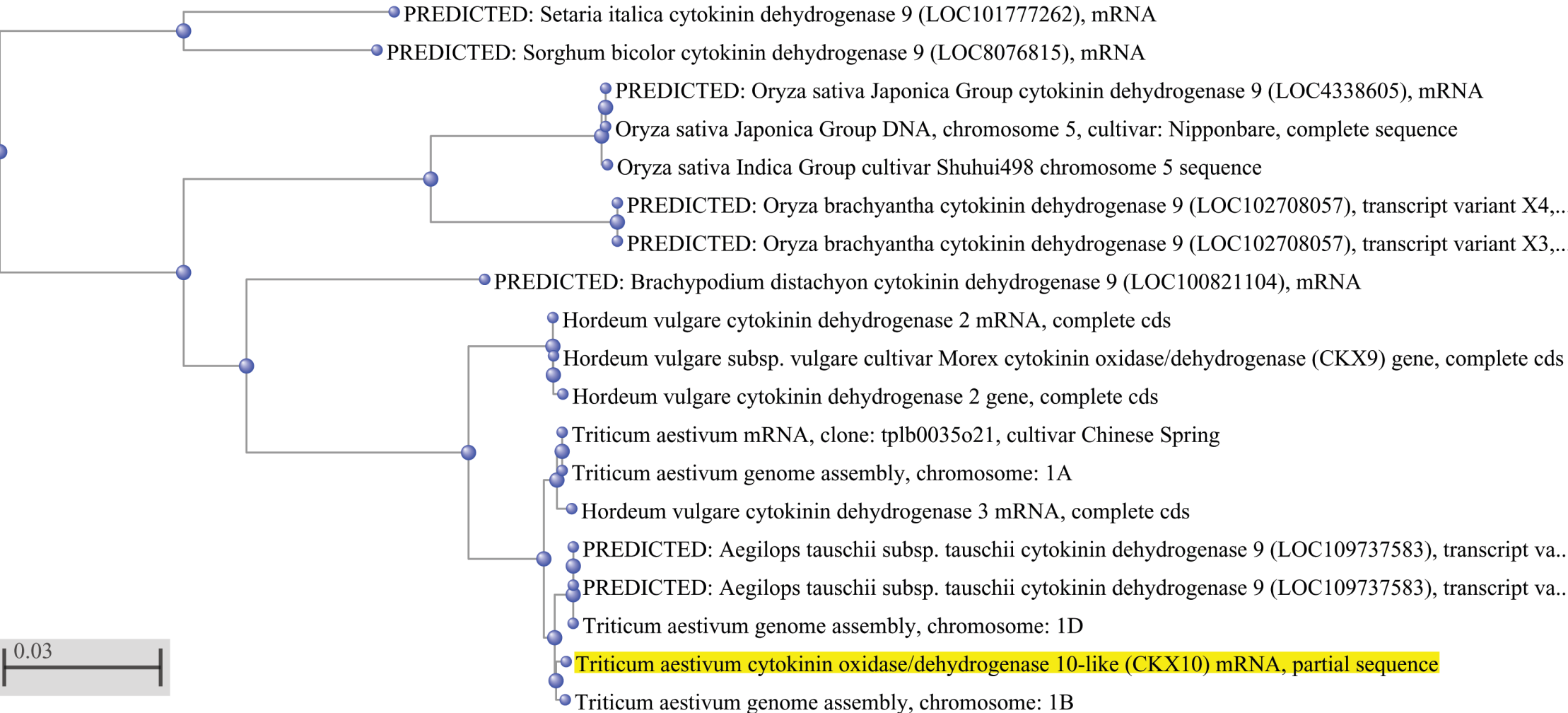

N

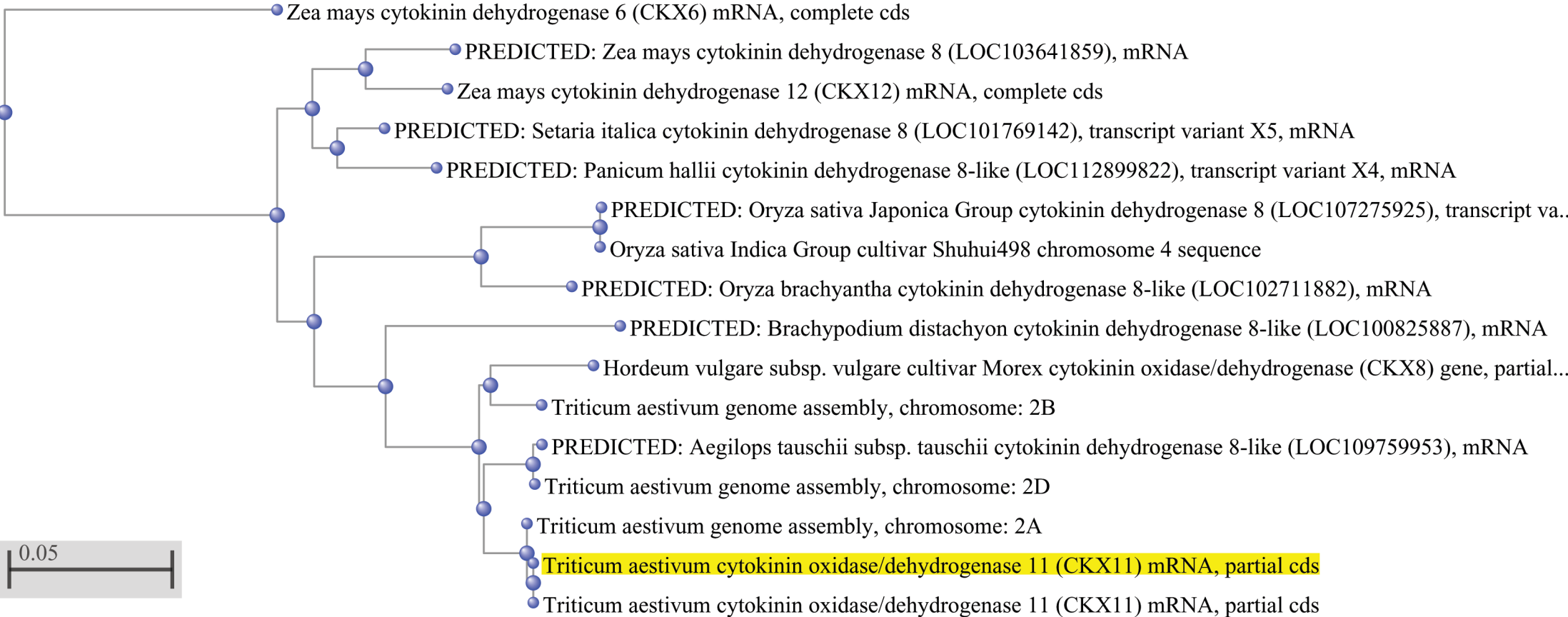

Supplement: S1 File — Figs A-N. The distance tree of pairwise comparison of TaCKX1 (A), TaCKX2.1 (B), TaCKX2.2 (C), TaCKX3 (D), TaCKX4 (E), TaCKX5 (F), TaCKX6 (G), TaCKX6-D1 (H), TaCKX6a02 (I), TaCKX7 (J), TaCKX8 (K), TaCKX9 (L), TaCKX10 (M), TaCKX11 (N) with their homologues and orthologues (queries are highlighted by yellow). (PDF) [file pone.0214239.s003.pdf]
